# Research on Shape–Performance Integrated Monitoring Technology for Planetary Gearboxes Based on the Integration of Artificial Intelligence, Finite Element Analysis, and Multibody Dynamics Simulation

**DOI:** 10.3390/s25185810

**Published:** 2025-09-17

**Authors:** Yanping Cui, Boshuo An, Zhe Wu, Ziao Shang, Xuanrui Zhang

**Affiliations:** School of Mechanical Engineering, Hebei University of Science and Technology, Shijiazhuang 050018, China; cuiyp@hebust.edu.cn (Y.C.); 15511381917@163.com (B.A.); 13930416196@163.com (Z.S.); r18633005956@163.com (X.Z.)

**Keywords:** planetary gearbox, morphology–performance integration, artificial intelligence, structural performance analysis, multibody dynamics analysis

## Abstract

To address gear tooth damage and the difficulty of acquiring performance data under high-speed and high-load operating conditions of planetary gearboxes, a digital twin-based system for operational state recognition and performance prediction is proposed, integrating morphological and functional characteristics. Driven by experimental data, the system incorporates finite element analysis, multibody dynamics simulation, artificial intelligence algorithms, and 3D visualization to achieve a virtual mapping of the gearbox’s geometric configuration, structural properties, and dynamic behavior. Structural performance is represented using finite element and dynamic simulation techniques combined with texture mapping, visualized through color gradients; dynamic performance is captured through multibody dynamics simulations and stored in a time-series database, presented as sequential images. The integrated system is constructed by combining a structural performance surrogate model, a system-driven model, and a dynamic performance database, enabling comprehensive functionality. Results demonstrate that the maximum error of the structural performance model is 3%, occurring only under specific working conditions, with negligible impact on the overall meshing performance evaluation of the sun gear. The maximum error in dynamic performance prediction is 1.68%, showing strong consistency with experimental data.

## 1. Introduction

With the continuous advancement of artificial intelligence and computer technology, intelligent manufacturing has become the core focus of global manufacturing development. Countries around the world are actively promoting its implementation to address the challenges brought by the new wave of industrial transformation [1]. The widespread application of next-generation information and communication technologies, as well as artificial intelligence technologies, is also driving traditional manufacturing industries toward intelligent manufacturing based on information technology [2].

Within the intelligent manufacturing system, the planetary gearbox, as one of the core transmission components, plays a crucial role in the efficient operation of equipment. It is widely used in aerospace [3], power equipment [4], military devices [5], and other fields, characterized by compact size, high load capacity, high transmission efficiency, and smooth power delivery [6,7]. However, planetary gearboxes also have certain drawbacks that pose risks to stable operation in practical applications. During prolonged high-load operation, gear meshing induces significant tooth surface contact stress and root bending stress, leading to gear wear and fracture, which adversely affects the smooth operation of the gearbox. Additionally, the complex design of planetary gearboxes often causes vibration and noise. These issues worsen under uneven loading or unstable working conditions, impacting overall performance and posing hidden risks to gearbox operation. Current research on the operational state diagnosis of planetary gearboxes primarily relies on vibration signal analysis, temperature monitoring, and other data acquisition techniques, supplemented by expert systems for state recognition. Traditional detection methods are limited by the closed structure of the gearbox and offline monitoring modes, resulting in certain latency and difficulty in timely capturing internal operational states and performance trends.

In 2009, the concept of the Digital Twin was clearly proposed by a U.S. Air Force laboratory as the “Airframe Digital Twin.” In 2010, NASA officially adopted the term “Digital Twin (DT)” in its reports on Modeling, Simulation, Information Technology, and Processing, as well as Materials, Structures, Mechanical Systems, and Manufacturing [8,9]. In 2011, the Air Force Research Laboratory (AFRL) suggested applying the Digital Twin to aircraft condition monitoring and life prediction [10]. FLORESCU et al. [11] proposed a digital twin modeling method to integrate intelligent machine tools and industrial robots within a flexible manufacturing system. The method enables simulation optimization from design to virtual commissioning and demonstrates its value in multi-device collaboration and system fault prediction. CUNHA et al. [12] proposed a modular digital twin modeling method for reconfigurable manufacturing systems, successfully applying it in smart factory environments. Chang et al. [13] introduced a digital twin-based cyber-physical fusion control platform, which, through a three-layer architecture and real-time bidirectional communication, enabled high-precision and low-latency synchronization control of industrial robots, significantly improving operational accuracy and interaction experience under complex tasks. Zhang et al. [14] developed a digital twin-based intelligent tool machining process modeling method, establishing an auxiliary equipment adaptive coupling mechanism and an enhanced tool wear identification model, realizing high-quality, low-energy intelligent machining under complex working conditions. Diogo Costa [15] proposed a human digital twin system based on a monocular camera and edge computing, using deep learning to estimate human posture and recognize object-picking tasks in industrial environments while preserving privacy.

The (Shape-Performance Integrated Digital Twin, SPI-DT) technology combines geometry and performance data to monitor critical components in enclosed structures, providing a new approach to addressing these challenges. Digital twin modeling initially relied primarily on physics-based methods, typically expressed in the form of mathematical equations. These modeling methods can be broadly categorized into experimental modeling and numerical modeling. However, with the emergence of large-scale data in digital twin environments, the proliferation of open-source advanced libraries (such as TensorFlow, Torch, and OpenAI), affordable computing infrastructure (CPU, GPU, and TPU), and high-fidelity training resources, data-driven modeling has gradually become mainstream. Between physics-based high-fidelity simulations and data-driven modeling, increasing research attention is being paid to their intersection. By transforming high-fidelity physics models into AI models and combining them with geometric data of physical objects, researchers have enabled low-order models to simulate full-order physical models effectively, trading some accuracy for much greater computational efficiency. RESON et al. [16] demonstrated that simulation data could support the operation of a digital twin, showing that such data can drive digital twin models. HAAG et al. [17], using a cantilever beam as the research object, simulated its deformation and stress distribution by integrating geometric models and finite element data, and visualized the deformation and stress results. S. Chakraborty et al. [18] proposed an approach leveraging Gaussian Process (GP) surrogate models, combined with discrete damped dynamic systems, to improve the modeling accuracy and adaptability of digital twin systems in data-sparse and noisy environments. Some studies [19] have conducted a systematic review of uncertainty quantification (UQ) methods for digital twins in the fields of intelligent manufacturing and robotics, summarizing the applications and challenges of techniques such as statistical inference, Bayesian methods, and stochastic sampling in improving model reliability and fidelity. Wang et al. [20] proposed a multi-fidelity moving least squares (MFS-MLS) framework that integrates numerical simulations and sensor data to achieve high-accuracy, real-time digital twin modeling and prediction, validating its effectiveness in truss structure testing. Lai et al. [21] proposed the SPI-DT approach, which integrates analytical models, numerical models, and AI models to enable efficient prediction and visual verification of the structural performance of critical components in heavy equipment. In wind turbine condition monitoring, a comprehensive strategy typically combines the monitoring of mechanical components with structural health monitoring of load-bearing structures. It relies on multiple non-destructive testing techniques to assess equipment under complex working conditions [22]. This approach similarly inspired the multi-source fusion digital twin modeling method proposed in this study for planetary gearboxes, which also holds potential for application in the condition monitoring of other critical equipment such as wind turbines and large-scale construction machinery.

Based on the analysis of the current research by the aforementioned scholars, the following key issues can be summarized:

(1) With improvements in digital twin model accuracy, the volume of processed data increases continuously, resulting in a gradual decline in model response speed.
(2) Existing studies mainly focus on structural performance variations at geometric model vertices. However, in high-speed and closed planetary gearbox applications, acquiring performance data is challenging. Relying solely on structural performance analysis cannot comprehensively reflect complex operational characteristics. In contrast, dynamic performance data are easier to obtain and provide more intuitive reflection of operational traits. Combining structural and dynamic performance aids in more accurately assessing planetary gearbox health and revealing performance under different operating conditions, providing reliable bases for intelligent evaluation.

Compared with the limitations of the above studies, this research proposes a digital twin modeling approach that deeply integrates finite element simulation, multibody dynamics simulation, artificial intelligence algorithms, and experimental data. This method enables multidimensional virtual mapping of the geometric configuration, structural performance, and dynamic behavior of planetary gearboxes, overcoming the limitations of accuracy and applicability caused by single-source modeling. This study employs color-gradient texture mapping to represent structural performance and time-series images to visualize dynamic performance. This approach achieves synchronized structural–dynamic mapping in a 3D visualization framework, allowing intuitive presentation of performance variations during the gear meshing process. Furthermore, by combining a structural performance surrogate model, a system-driven model, and a dynamic performance database, a closed-loop digital twin system is established that integrates data acquisition, model prediction, and performance visualization. This system realizes real-time monitoring of operational state recognition and performance prediction of the gearbox, providing a novel approach for fault warning and performance optimization under complex working conditions.

Planetary gearboxes, as core components of mechanical transmission systems, often operate under high-speed and high-load conditions. These conditions make gear teeth prone to damage and make it difficult to acquire operational and performance data, severely affecting safe and stable operation. Based on this, the research question of this study is: Can the proposed digital twin model effectively identify typical faults of planetary gearboxes and play a role in performance prediction? To address this question, this paper proposes a shape–performance integrated digital twin technology, and develops a data-driven method to detect operational state and performance variations in planetary gearboxes.

## 2. Overall Design Scheme of the Morphology–Performance Integrated System for Planetary Gearboxes

### 2.1. Digital Twin Technology for Morphology Integration of Planetary Gearboxes

Morphology–performance integrated digital twin technology is a key approach applied in mechanical system design and condition monitoring. Its core lies in overcoming the traditional separation between “geometric morphology” and “performance analysis” by employing multi-physical field coupled modeling, data fusion, and optimization algorithms to achieve collaborative representation and dynamic interaction between geometry and performance. This enables each position in the geometric model to be associated with and obtain corresponding performance information. The technology framework holds significant application potential in equipment manufacturing and intelligent operation and maintenance.

### 2.2. System Architecture of the Morphology–Performance Integrated System for Planetary Gearboxes

Building upon morphology–performance integrated digital twin technology, this study further investigates the establishment of a morphology–performance integrated system for planetary gearboxes. Specifically, the core design objectives of the system—including modeling and simulation, data acquisition, state assessment, and system interaction—should be clearly defined. By constructing a scientifically sound technical framework and integrating diverse data sources, the system aims to fuse data-driven approaches with artificial intelligence, thereby developing an efficient and accurate morphology–performance integrated platform to enhance the safety and stability of planetary gearboxes.

#### 2.2.1. System Design Objectives and Technical Framework

System Design Objectives: Taking the planetary gearbox as the target, this study aims to construct dual digital twin models of “morphology” and “performance.” Through geometric feature modeling and model reconstruction, it achieves accurate restoration and visual representation of the geometric morphology. To realize the integration of “performance” datasets, structural performance analysis and dynamic performance analysis are conducted to build surrogate performance models and databases. Driven by experimental data, the morphology–performance integrated system is established to enable real-time operation state recognition, performance prediction, and anomaly warning functions, thereby ensuring the safe and stable operation of the planetary gearbox.

**Figure 1:**
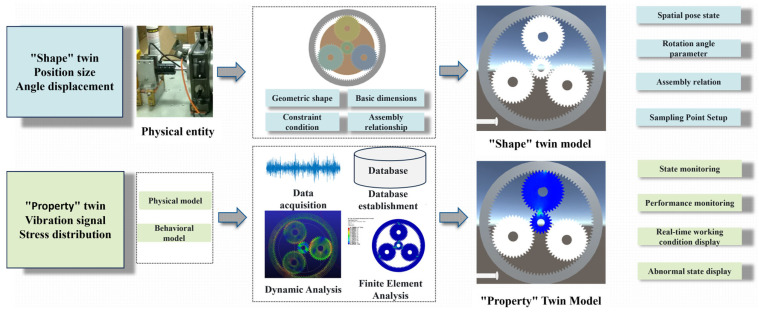
Technical Framework of the Morphology–Performance Integrated System for Planetary Gearboxes.

Figure 1 illustrates the technical framework of the morphology–performance integrated system for planetary gearboxes. In the development of this digital twin-based system, the first step is to acquire geometric data of the planetary gearbox and establish its model. The planetary gearbox model construction is based on geometric structures and assembly constraints, combined with fundamental parameters of key components such as the sun gear, planet gears, internal ring gear, and planet carrier. This model not only includes the spatial distribution of each gear tooth and its shaft system but also reflects assembly constraints, ensuring that the 3D model remains geometrically and structurally consistent with the physical entity. To enable visualization and interaction of the “morphology” model within Unity, the completed 3D model is discretized and imported into the Unity environment. This allows users to directly drag and inspect the hierarchical structure and geometric appearance of the planetary gearbox within Unity scenes. Subsequently, by analyzing the spatial pose states and rotational angles of each gear tooth, the system extracts and assigns pose and angle information to the gear model, ensuring accuracy in geometric shape, assembly relationships, and rotation angles.

In the “performance” twin phase, the planetary gearbox’s geometric model is imported into finite element and multibody dynamics simulation software. Structural analyses are then conducted on key components, such as the sun gear, planet gears, and internal ring gear, as well as on overall dynamic performance. Finite element analysis is first used to evaluate the structural performance of each gear tooth under the same speed and load conditions but different sun gear fault scenarios, identifying critical risk areas or failure-prone gear teeth. Multibody dynamics analysis then simulates the meshing process, load distribution, and dynamic characteristics of the planetary gearbox to analyze its dynamic behavior during gear transmission. Subsequently, finite element data of fault-prone gear teeth, dynamic analysis data, and experimental data are extracted. These data are combined with artificial intelligence algorithms to train structural performance surrogate models and morphology–performance integrated system driving models, establishing a dynamic performance database for the planetary gearbox. Finally, the integrated system visualizes actual operational input data alongside real-time structural and dynamic performance results, completing the operation of the morphology–performance integrated system.

The morphology-level twin continuously updates spatial poses, assembly relationships, and rotation angles in real time. Meanwhile, the performance-level twin dynamically assesses the planetary gearbox’s operational state from experimental signals and predicts its structural and dynamic performance. The morphology–performance integrated system plays a critical role in the monitoring and maintenance of planetary gearboxes, providing essential safety assurance for equipment manufacturing and industrial applications.

#### 2.2.2. Layered Implementation Architecture

**Figure 2:**
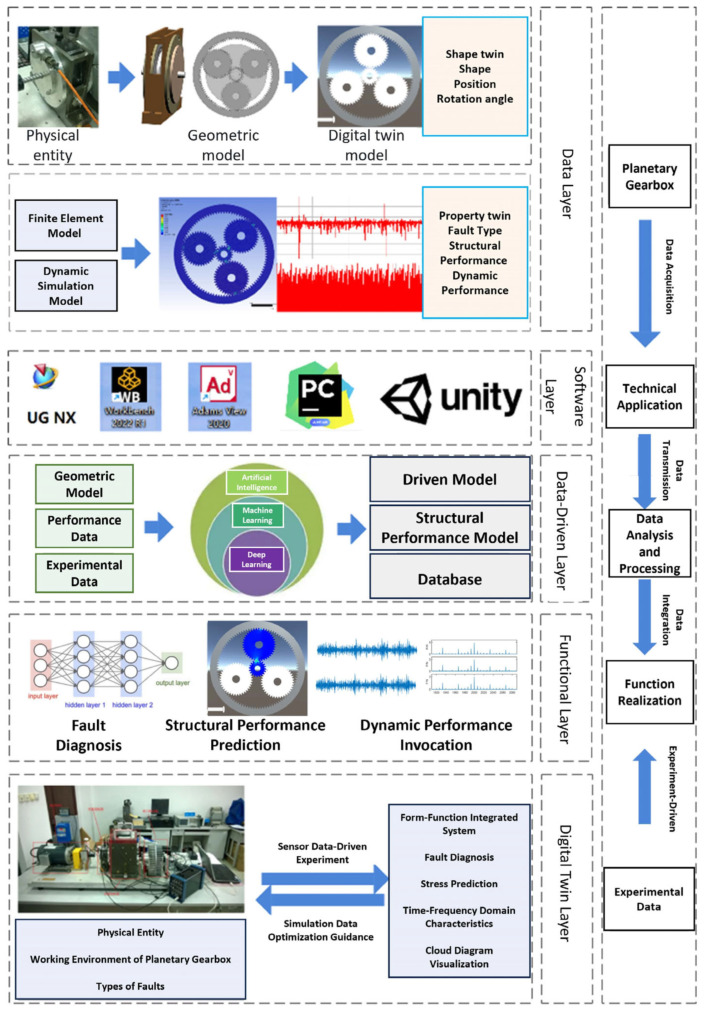
Overall Architecture of the Morphology–Performance Integrated System for Planetary Gearboxes.

Based on the aforementioned twin model design, the morphology–performance integrated system architecture is developed to enable operation state recognition, performance prediction, and key component visualization of the planetary gearbox. Figure 2 illustrates the overall system architecture, which consists of the data layer, software layer, data-driven layer, functional layer, and twin layer.

The morphology–performance integrated system for planetary gearboxes provides solutions for diverse needs, including operation state recognition, performance analysis, and interactive visualization. It achieves this through collaborative interaction among the data, software, data-driven, functional, and digital twin layers.

## 3. Structural Performance Analysis and Surrogate Modeling of Planetary Gearboxes Under Morphology–Performance Collaboration

### 3.1. Finite Element Analysis of the Structural Performance of the Planetary Gearbox

**Figure 3:**
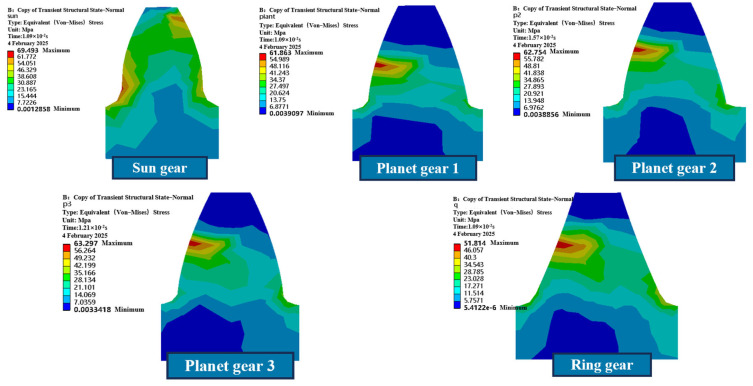
Maximum Stress Cloud Map of Each Gear Tooth in the Planetary Gearbox.

Figure 3 shows the maximum stress cloud diagram of each gear tooth in the planetary gearbox. As can be seen, the maximum equivalent stresses of the sun gear, planet gear 1, planet gear 2, planet gear 3, and the ring gear are 69.493 MPa, 61.863 MPa, 62.754 MPa, 63.287 MPa, and 51.814 MPa, respectively, all of which are below the yield strength of 45# steel (355 MPa). The transient stress analysis indicates that the sun gear, as the driving component, exhibits the maximum stress value of 69.493 MPa, concentrated on the back side of the meshing tooth surface, implying that it is subjected to significant bending stress. This phenomenon is closely related to root stress concentration during torque transmission. The maximum stresses of the planet gears are distributed in the meshing tooth surface regions, with values of 61.863 MPa, 62.754 MPa, and 63.287 MPa, respectively, showing that the load is relatively evenly distributed, with small differences among the three and a consistent overall stress trend. The maximum stress of the ring gear is 51.814 MPa, also concentrated in the tooth surface contact region, with a relatively low stress level.

According to the above patterns, the maximum equivalent stress at the root of the sun gear in the planetary gearbox is significantly higher than that of the other gears. As the driving gear, it bears the task of transmitting driving force and represents a weak point in the overall structure. The maximum stress of the three planet gears occurs during single-tooth engagement, showing consistent distribution characteristics with slight differences in timing, indicating a balanced load distribution among them and preventing overloading. The maximum stress of the sun gear, planet gears, and internal ring gear all occurs immediately after the rotational speed and torque have been applied. At this moment, although the external load has stabilized, the system inertia induces short-term stress concentration, and the stress gradually decreases outward along the contact area, in accordance with the principles of contact mechanics.

**Figure 4:**
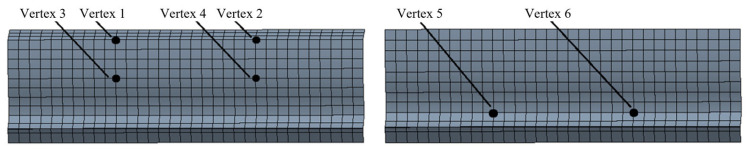
Selected Vertex Positions on the Sun Gear Contact Surface.

**Figure 5:**
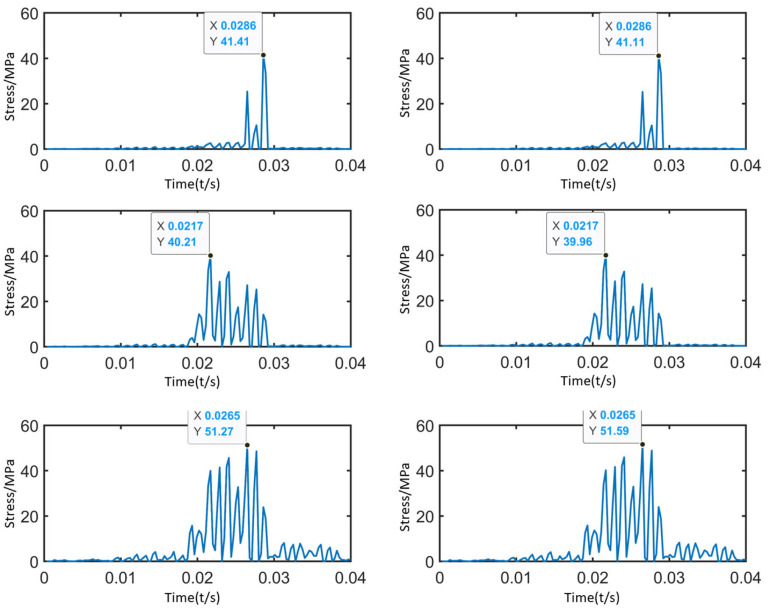
Stress–Time Curves of the Vertices.

The rotational speed and load are fully applied after 0.01 s, and the contact tooth surfaces at the stable stage are selected for analysis. Figure 4 shows the positions of the vertices on the sun gear contact surface. Six vertices are selected along the same axial direction on each contact tooth surface, including the tooth tip, the contact line, and the tooth root on the opposite side of the contact surface. Figure 5 shows the stress–time curves of each vertex. From the comparative analysis of the stress variations at the sun gear tooth tip, contact line, and root, it can be observed that:

The stress curves of the three pairs of vertices—tooth tip (vertices 1 and 2), contact line (vertices 3 and 4), and tooth root (vertices 5 and 6)—show only minor differences. The symmetrical structure of the gear along the tooth width ensures uniform load distribution, and no local load concentration occurs during meshing. The stress amplitude and fluctuation trends are consistent for vertices on both sides of the same position. The peak stress at the sun gear tooth tip is 41.41 MPa, at the contact line is 40.21 MPa, and at the tooth root is 51.59 MPa. In this planetary gearbox, the stress at the sun gear tooth root is higher than that at the tooth tip and contact line. Under actual loading conditions, gear tooth failure or fracture is likely to occur at the sun gear tooth root. Since the sun gear is prone to failure, this study primarily focuses on the stress distribution of the sun gear.

### 3.2. Multi-Fault Stress Field Analysis of Planetary Gearboxes

**Figure 6:**
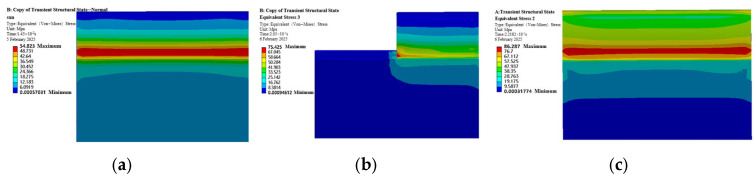
Stress Cloud Diagram of the Sun Gear under Normal and Various Fault Conditions. (**a**) Normal Condition. (**b**) Tooth Breakage Condition. (**c**) Wear Condition.

Figure 6 shows the stress cloud maps of the sun gear under normal, tooth breakage, and wear conditions. Under normal operation, the maximum equivalent stress of the sun gear is 54.823 MPa. When a tooth breakage fault occurs, the maximum stress rises to 75.425 MPa, an increase of 37.6% compared to the normal state. Under wear fault conditions, the maximum stress further increases to 86.287 MPa, representing a 57.3% increase. These results indicate that fault conditions exacerbate the maximum equivalent stress of the sun gear. The stress distribution characteristics are as follows. In the normal state, the equivalent stress shows a gradient along the tooth surface, uniformly diffusing from the root to the meshing surface, with overall low stress levels. During tooth breakage, the remaining half tooth bears the entire original load, causing localized stress increases, although effective meshing between the sun gear and planet gears is maintained. In the wear fault condition, the meshing contact position shifts, and the stress peak timing moves from conventional tooth surface meshing to the meshing between the sun gear tooth tip and the planet gear surface, leading to increased stress amplitude in the worn sun gear.

### 3.3. Data-Driven Structural Performance Surrogate Model Development

**Figure 7:**
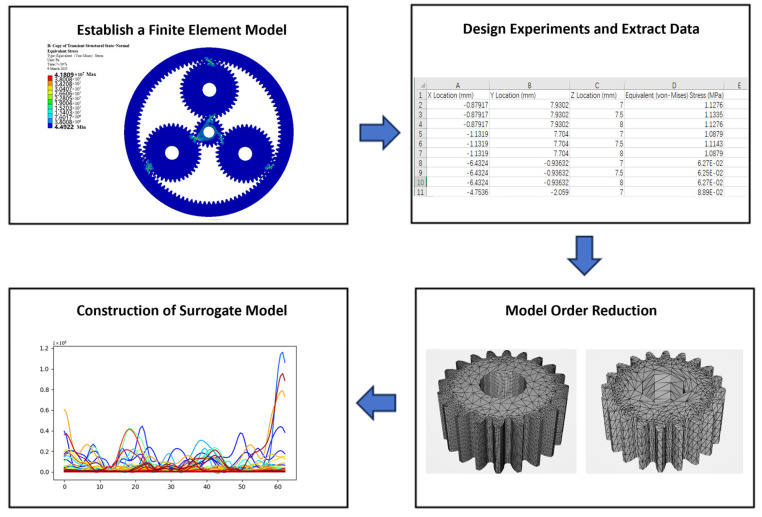
Structural Performance Model Development Process for Planetary Gearboxes.

Figure 7 illustrates the development process of the structural performance model for the planetary gearbox. After completing finite element simulations and obtaining structural performance data, the meshing process of a single sun gear tooth is selected as the study condition. Based on this, an artificial intelligence algorithm script is developed in Python to construct a structural performance surrogate model, enabling accurate prediction of stress evolution throughout the entire meshing process of the gear tooth.

The specific development process is as follows:

(1) Experiment Design

Based on historical operational data of the planetary gearbox, the sun gear’s rotational speed and the planet carrier’s load within a single meshing cycle are determined. After applying appropriate boundary conditions, finite element simulations are conducted to obtain stress data for the entire meshing process of a specific tooth on the sun gear during its steady-state phase. A total of 50 frames representing different meshing angles are selected, with stress data from all vertices extracted for each frame. These are used as working conditions to ensure full coverage from initial to final meshing contact.

(2) Model Order Reduction

**Figure 8:**
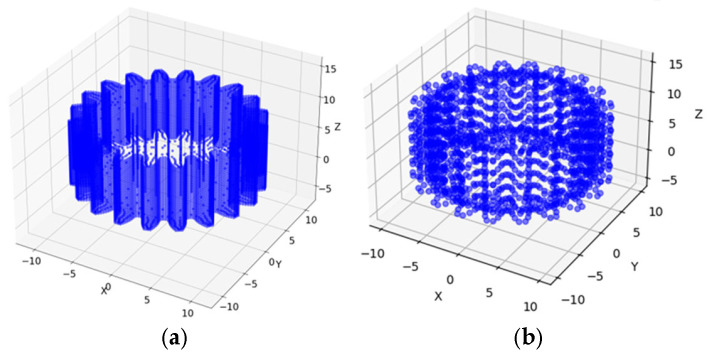
Point Cloud Model Order Reduction. (**a**) Pre-Reduction Model. (**b**) Post-Reduction Model.

The sun gear is discretized using a coarse mesh. Figure 8 presents the vertex model before and after reduction. A Python script is employed to process the vertices in the files, ultimately exporting unique vertex coordinates and their corresponding indices to improve system response time. The vertex deduplication improves data access efficiency, while the index retains the connectivity of all vertices before reduction.

(3) Structural Performance Surrogate Model Construction

By introducing the (Radial Basis Function, RBF) neural network framework, an artificial intelligence model is constructed to meet real-time prediction requirements. As a typical feedforward neural network, the RBF network exhibits unique advantages in function approximation. Its core feature lies in a three-layer topology composed of multiple kernel functions and a linear output layer, which improves training efficiency and generalization capability compared with traditional neural networks.

**Figure 9:**
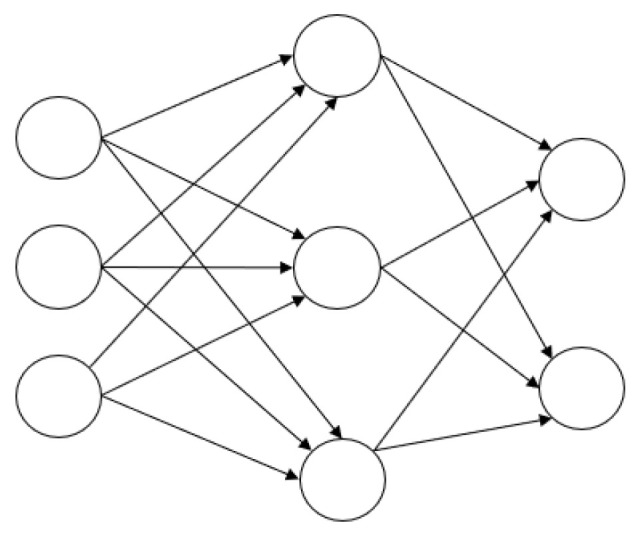
Gaussian-Kernel-Based RBF Neural Network.

The RBF neural network uses the Gaussian function as its kernel, and the structure of the Gaussian-kernel-based RBF network is shown in Figure 9. The first column represents the input layer, which consists of the vertex data to be predicted; this layer primarily receives input data without processing it. The second column is the hidden layer, where the neurons use Gaussian functions, allowing the model to automatically recognize the number of discretized gear vertices. Each vertex is nonlinearly mapped from the input space through the Gaussian kernel function. The third column is the output layer, which employs a linear activation function to perform a weighted integration of the hidden layer information, producing the final output of the RBF neural network.

The transfer function of this neural network is the Gaussian kernel function, as shown in Equation (1):

(1) $$\varphi_{i}\left(t\right)=e^{-{\left(\frac{t}{\delta }\right)}^{2}}$$

In the equation: t—the distance between the input data and the vertex center;

δ—the width parameter of the Gaussian function, which controls its smoothness;

$\varphi_{i}\left(t\right)$—the output of the *i*-th vertex, i.e., the activation value of that vertex.

$\varphi_{i}\left(t\right)$ represents the similarity between the input parameter and the vertex center. The greater the distance between the input data and the vertex center, the lower the similarity and the smaller its impact on the output; conversely, a closer distance leads to a larger output from the hidden layer. Therefore, this network possesses strong approximation capability, with significant influence exerted only by local vertices.

Once the transfer function is determined, the model can be trained using the finite element simulation data of the planetary gearbox. The training is divided into two stages: determining the weights $C_{j}$ and $D_{j}$ between the first and second layers, and determining the weights $W_{j}$ between the second and third layers. The training process is as follows:

Definition of the input vector X and the output vector Y

$X={\left[x_{1},x_{2},\cdots ,x_{n}\right]}^{T}$ is the input layer vector, where n is the dimension of the vector, i.e., the number of units in the input layer.

$Y={\left[y_{1},y_{2},\cdots ,y_{q}\right]}^{T}$ is the output layer vector, where q is the number of units in the output layer.

Initialization of Weights and Width Vectors

The initialization of weights involves setting initial parameters for the connections between the layers of the neural network according to specific rules, in order to optimize the training efficiency and convergence speed of the model:

(2) $$W_{k}={\left[W_{k1},W_{k2},\cdots ,W_{kp}\right]}^{T},k=\left(1,2,\cdots ,q\right)$$

(3) $$W_{kj}=mink+j\frac{maxk-mink}{q+1},j=\left(1,2,\cdots ,p\right)$$

In the equation, mink—the minimum value of the *k*-th output unit in the input set; and maxk—the maximum value of the *k*-th output unit in the input set.

The initialization of the width vector $d_{ji}$ controls the response intensity of a neuron to information near its center, as shown in Equation (4):

(4) $$d_{ji}=d_{f}\sqrt{\frac{1}{N}\sum_{k=1}^{N}\left(x_{i}^{k}-c_{ji}\right)}$$

In the equation, $d_{f}$—the width adjustment factor, which affects the response capability of the RBF neural network.

Hidden Layer Output

The hidden layer computes the output $z_{j}$ of each neuron by calculating the distance between each input signal and the neuron, and then applying the Gaussian function:

(5) $$z_{j}=exp\left(-{\left\|\frac{X-C_{j}}{D_{j}}\right\|}^{2}\right),j=\left(1,2,\cdots ,p\right)$$

In the equation: $C_{j}$—the center vector of the *j*-th neuron;

X—the input vector;

$D_{j}$—the width vector of the *j*-th neuron.

Output Layer Calculation

The output of the neurons in the output layer is obtained by the weighted summation of the hidden layer outputs. The input to the output layer is expressed as in Equation (6):

(6) $$Y={\left[y_{1},y_{2},\cdots ,y_{q}\right]}^{T}$$

(7) $$y_{k}=\sum_{j=1}^{p}w_{kj}z_{j},k=\left(1,2,\cdots ,q\right)$$

In the equation, $w_{kj}$—the weight between the hidden layer and the output layer.

**Figure 10:**
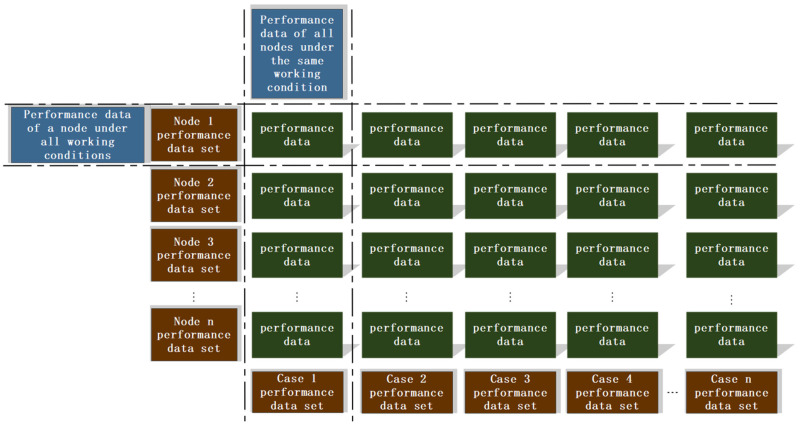
Surrogate Model Structure Composition.

The K-nearest neighbors (KNN) algorithm is applied to interpolate the stress data of the reduced mesh vertices, addressing the low precision caused by coarse meshing. A RBF neural network with a Gaussian kernel is then used to train the structural performance surrogate model, enabling accurate prediction of stress distribution throughout the full meshing process of a single sun gear tooth. The input of the surrogate model consists of working condition data at different meshing angles (frames), while the output is the predicted stress values at each vertex. As shown in Figure 10, each vertex is fitted with a numerical expression based on all available working conditions. The prediction scope of this model is limited to the meshing process of a specific sun gear tooth. If predictions go beyond this range, underfitting or overfitting may occur, necessitating additional data to cover a wider domain.

### 3.4. Analysis of Surrogate Model Prediction Results

To validate the effectiveness of the structural performance model, finite element simulation results and surrogate model predictions of the sun gear under different fault conditions were compared. Simulations were conducted for typical fault types, including normal sun gear operation, tooth breakage, and wear during the meshing process. The surrogate model was evaluated using metrics such as the coefficient of determination (R^2^) and root mean square error (RMSE). Additionally, meshing process curves at selected vertices were plotted alongside real-time surrogate model predictions for comparative analysis.

Table 1 presents the surrogate model evaluation results for a selected working condition under the normal state of the sun gear. One hundred vertices were randomly chosen from this condition for assessment. The comparison methods include RBF, Gaussian Process Regression (GPR), and Random Forest (RF).

According to the data in Table 1, both the RBF and GPR surrogate models achieve accuracy greater than 0.8, which generally indicates acceptable surrogate modeling performance [23]. Among them, the RBF model demonstrates higher accuracy, with a coefficient of determination exceeding 0.9, and lower RMSE and MAE values. To enhance model accuracy, the RBF method is selected for constructing surrogate models of the sun gear under various operating conditions.

Table 2 presents the evaluation results of the surrogate model under different operating conditions of the sun gear for a selected working condition. Based on the evaluation metrics of the surrogate models in the normal, tooth breakage, and wear states, it is evident that the RBF surrogate model consistently maintains a coefficient of determination (R^2^) above 0.9 across all conditions, indicating strong fitting capability and high prediction accuracy. In the wear condition, the root mean square error (RMSE) and mean absolute error (MAE) reach 2.1 MPa and 0.9 MPa, respectively—the highest among the three operating states. However, considering the maximum equivalent stress of 86.287 MPa obtained from the finite element analysis, the surrogate model’s error remains relatively small. This demonstrates that the RBF model significantly improves computational efficiency while maintaining satisfactory accuracy.

**Figure 11:**
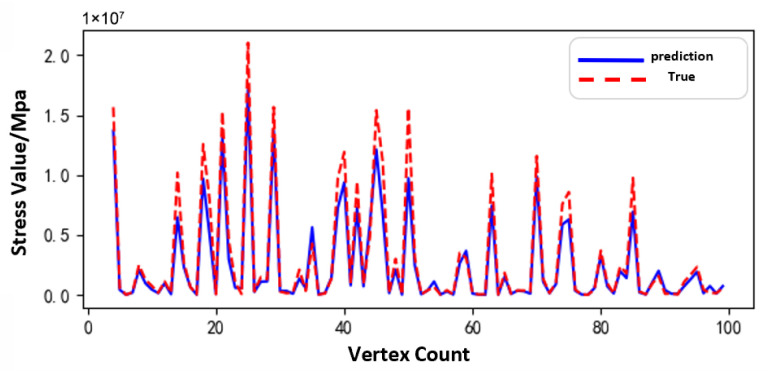
Comparison of Sampled Results under Normal Condition of the Sun Gear.

**Figure 12:**
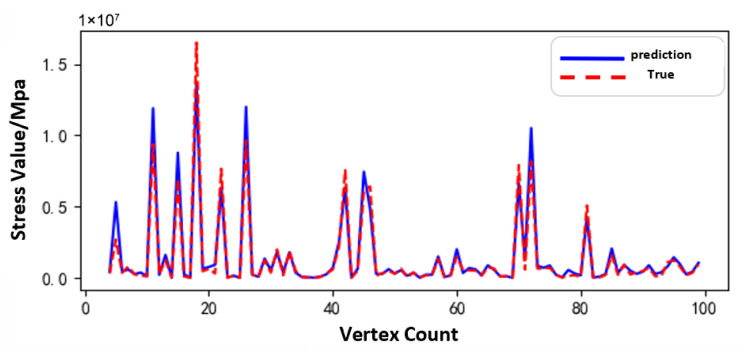
Comparison of Sampled Results under Tooth Breakage Condition of the Sun Gear.

**Figure 13:**
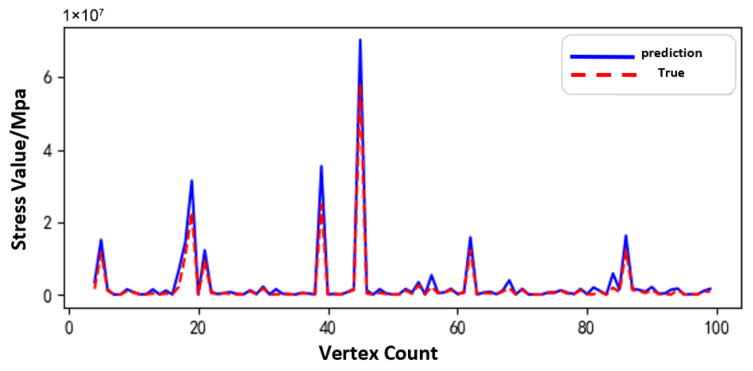
Comparison of Sampled Results under Wear Condition of the Sun Gear.

Figure 11, Figure 12 and Figure 13 show the prediction results of the surrogate model for 100 sampled vertices under different operating conditions of the sun gear in a selected working condition. The predicted values closely match the actual values overall, indicating that the surrogate model possesses strong predictive capability.

Based on the surrogate model’s vertex sampling predictions and corresponding curves, the RBF surrogate model accurately captures both the magnitude and variation trends of stress under the normal, tooth breakage, and wear conditions. This demonstrates its strong adaptability across different operating states of the planetary gearbox. Compared to purely data-driven approaches, the surrogate model effectively approximates the full-order model using a limited dataset, reducing data storage requirements while maintaining high prediction accuracy. Thus, it provides an efficient and reliable tool for establishing the morphology–performance integrated system.

## 4. Dynamic Performance Analysis and System Driving Model of Planetary Gearboxes

### 4.1. Analysis of Dynamic Simulation Results

(1) Angular Velocity Analysis

**Figure 14:**
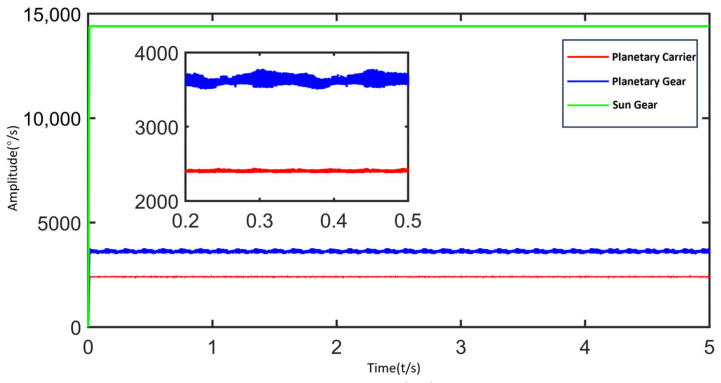
Rotational Speeds of Gear Teeth in the Planetary Gearbox.

Angular velocity comparison can be used to validate the model. According to calculations, the theoretical angular velocities are 14,400°/s for the sun gear, 3600°/s for the planet gears, and 2400°/s for the planet carrier. As shown in Figure 14, the green, blue, and red curves represent the time histories of the sun gear, planet gears, and planet carrier, respectively. Due to the use of a step function loading method, the initial 0–0.01 s in the simulation corresponds to the speed ramp-up phase. During this phase, the input sun gear speed gradually increases from 0 to the stable value of 14,400°/s (equivalent to 2400 rpm). From 0.01 s to 5 s is the steady-state operation phase. The average angular velocity during this stable phase is selected as the evaluation metric for the system’s steady-state response.

Table 3 presents the theoretical and simulated angular velocities of the sun gear, planet gears, and planet carrier. In this simulation, the errors for the planet gears and planet carrier are 0.087% and 0.099%, respectively. Overall, the errors are minimal, with deviations between simulation and theoretical results within 0.1%, demonstrating the accuracy of the rigid-flexible coupled dynamic model of the planetary gearbox.

(2) Contact Force Analysis

Table 4 lists the notation of frequencies and their theoretical amplitudes, serving as a reference for frequency domain feature analysis and simulation validation.

**Figure 15:**
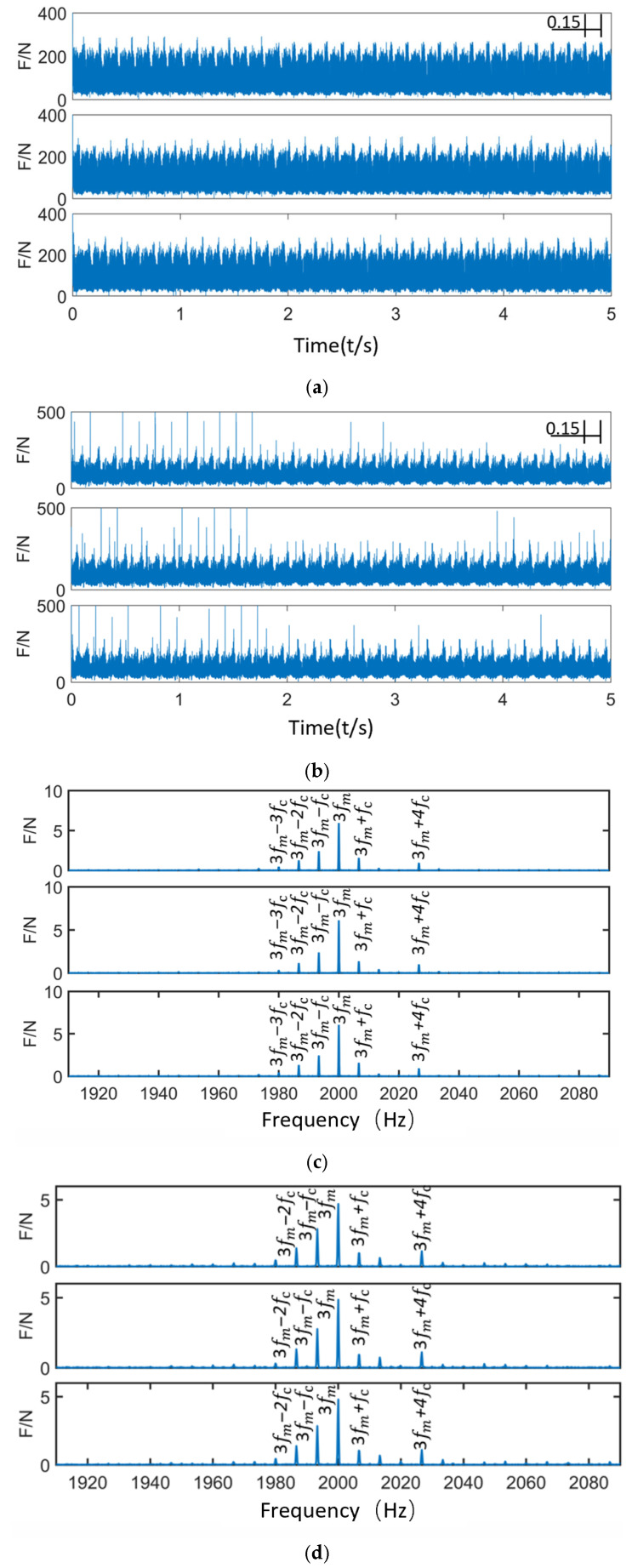
Time-Domain and Frequency-Domain Diagrams of Contact Force for the Sun Gear under Normal Operating Conditions. (**a**) Time-Domain Diagram of Sun Gear–Planet Gear Contact Force. (**b**) Time-Domain Diagram of Planet Gear–Ring Gear Contact Force. (**c**) Frequency-Domain Diagram of Sun Gear–Planet Gear Contact Force. (**d**) Frequency-Domain Diagram of Planet Gear–Ring Gear Contact Force.

Figure 15 presents the time-domain and frequency-domain contact force diagrams of the planetary gearbox under normal operating conditions. In the time-domain plot of the sun gear–planet gear contact force, the contact signals between the sun gear and the three planet gears are evenly distributed, with no evident impacts. The contact force curves exhibit periodic variation, with a cycle period of 0.15 s—corresponding to one full revolution of the planet carrier (i.e., the revolution of the planet gears). This indicates a planet carrier rotational frequency of 6.67 Hz, consistent with the theoretical value. In the time-domain plot of the planet gear–ring gear contact force, the trend is similar to that of the sun–planet gear contact. Some regions show slightly higher amplitudes than those of the sun–planet contact, but this has minimal impact on the overall time-domain signal. In the frequency-domain plots, the contact force frequency components for the sun–planet and planet–ring gear pairs are shown. They are mainly concentrated around the meshing frequency and the modulation band caused by the planet carrier. The dominant frequency components are distributed between $3f_{m}$ and $f_{c}$, confirming that the contact force variation is modulated by the time-varying meshing path (planet carrier rotation). These observations indicate that the system is operating stably, and the meshing condition is sound.

Analysis of the sun gear’s contact force in both time and frequency domains under different operating conditions reveals that, in the normal state, the contact force distribution exhibits strong regularity with stable amplitude fluctuations. The frequency spectrum is primarily concentrated at the meshing frequency and its harmonics, validating the effectiveness of the established rigid-flexible coupled dynamic model that accounts for time-varying effects.

(3) Vibration Characteristics Analysis

**Figure 16:**
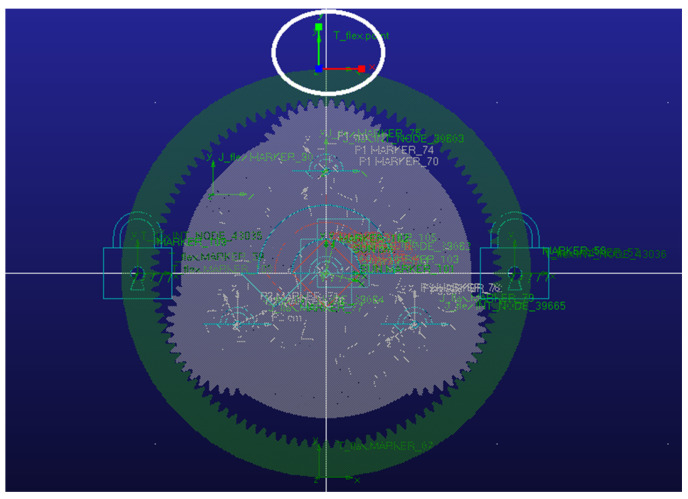
Measurement Point Locations of Vibration Signals in the Rigid–Flexible Coupled Dynamic Model of the Planetary Gearbox.

Figure 16 shows the measurement point locations where the vibration signals in the y-direction are extracted for analyzing the vibration characteristics of the planetary gearbox.

**Figure 17:**
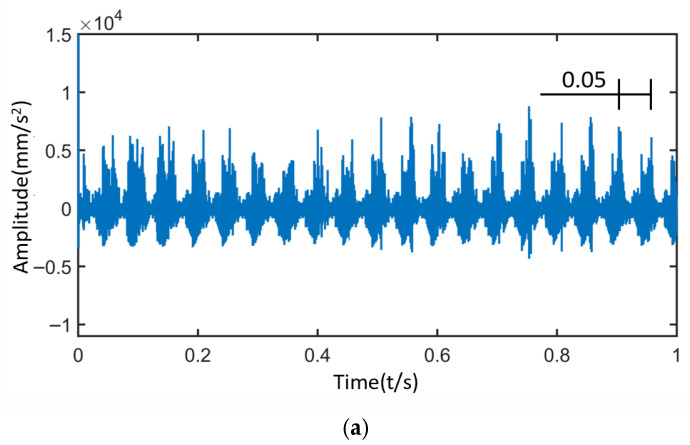

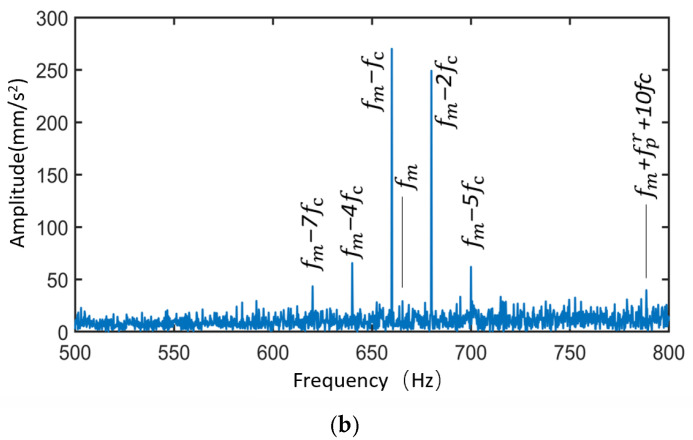
Time-Domain and Frequency-Domain Signals of the Sun Gear under Normal Operating Condition. (**a**) Time-Domain Signal. (**b**) Frequency-Domain Signal.

Figure 17 shows the time-domain and frequency-domain signals of the sun gear under normal operating conditions. When the planetary gearbox is operating normally, due to the planet gear passing effect, the signal at the measurement point exhibits three distinct modulations within one rotation period of the planet carrier (0.15 s). The amplitude of each modulation is relatively consistent, resulting in frequency-domain modulation primarily occurring at three times the planet carrier rotational frequency.

According to Figure 17, the frequency spectrum exhibits the following key characteristics:

① The dominant sidebands in the frequency spectrum are located at positions such as $f_{m}-7f_{c}$, $f_{m}-4f_{c}$, $f_{m}-f_{c}$, $f_{m}+2f_{c}$ and $f_{m}+5f_{c}$ corresponding to the general form $f_{m}-f_{c}\pm \mathrm{Na}f_{c}$. During the gear meshing vibration modulation process, the modulation effect of the planet carrier plays a dominant role.

② The dominant sideband spacing is $Nf_{c}$, where *N* represents the number of planet gears. This indicates that under normal operating conditions, the meshing vibration impacts between the sun gear and each planet gear are highly consistent, and the meshing interactions among the planet gears exert a periodic influence on the vibration characteristics.

③ The frequency spectrum also shows the presence of a frequency band at $f_{m}+f_{p}^{r}+10f_{c}$, indicating the existence of certain geometric errors in the planet gears, which may lead to distributed faults. (Note: *N* = 3, a, b, d = 0, 1, 2, …).

The simulation results indicate that under normal operating conditions, the gear meshing vibration of the planetary gearbox is influenced by the modulation effect of the planet gears, and its time-domain and frequency-domain characteristics exhibit clear periodicity and regularity. However, due to geometric or assembly errors, distributed faults may still occur in the planetary gearbox.

**Figure 18:**
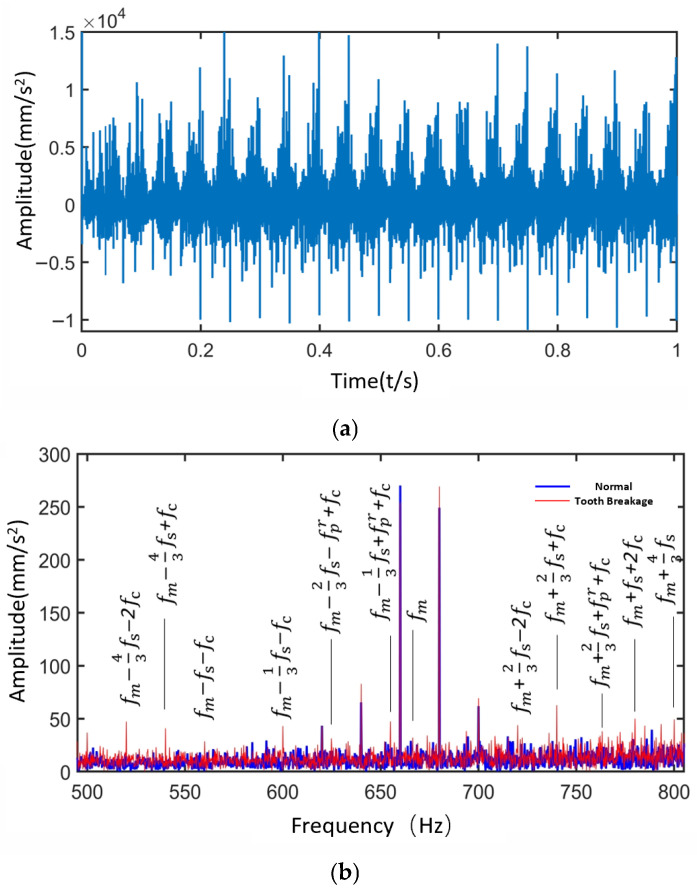
Time-Domain and Frequency-Domain Signals of the Sun Gear under Tooth Breakage Condition. (**a**) Time-Domain Signal of the Sun Gear under Tooth Breakage Condition. (**b**) Frequency-Domain Signals of the Sun Gear under Normal and Tooth Breakage Conditions.

**Figure 19:**
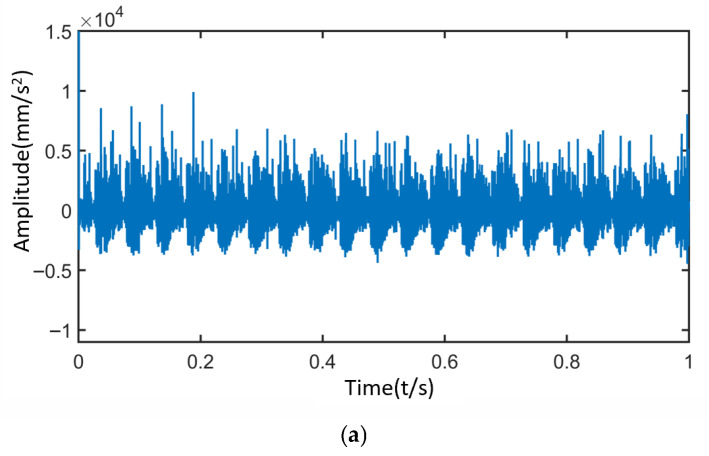

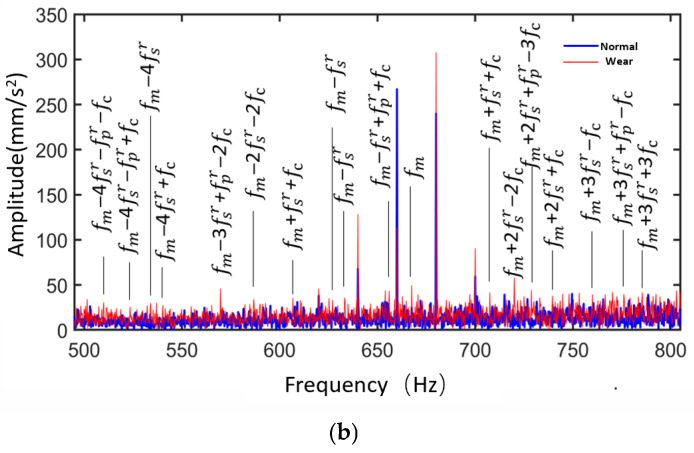
Time-Domain and Frequency-Domain Signals of the Sun Gear under Wear Condition. (**a**) Time-Domain Signal of the Sun Gear under Wear Condition. (**b**) Frequency-Domain Signals of the Sun Gear under Normal and Wear Conditions.

Figure 18 and Figure 19 present the time-domain and frequency-domain signals for the sun gear under local fault–normal and distributed fault–normal conditions, respectively.

① Tooth breakage is classified as a local fault. Compared with the normal state, the frequency spectrum under local fault conditions exhibits numerous sidebands related to the local fault frequencies, such as $f_{m}\pm af_{s}$ and $f_{m}\pm af_{s}\pm bf_{c}$. $f_{m}-f_{s}-f_{c}$, $f_{m}+f_{s}+2f_{c}$ band, etc., appear in the figure.

② In the case of local faults in the sun gear, differences in impact responses during meshing with the three planet gears lead to the emergence of sidebands such as $f_{m}\pm \frac{a}{N}f_{s}$, $f_{m}\pm \frac{a}{N}f_{s}\pm bf_{c}$, and $f_{m}\pm \frac{a}{N}f_{s}\pm bf_{c}\pm df_{p}^{r}$—for example, $f_{m}-\frac{1}{3}f_{s}-f_{c}$ and $f_{m}-\frac{2}{3}f_{s}-f_{p}^{r}+f_{c}$ appear in the frequency spectrum.

③ The wear fault of the sun gear is classified as a distributed fault. The frequency spectrum shows numerous sidebands related to distributed fault frequencies, mainly concentrated around frequency regions such as ($f_{m}\pm af_{s}^{r}\pm bf_{p}^{r}\pm df_{c}$).

Based on the above observations, it can be concluded that when the sun gear is in normal, local fault, and distributed fault states, the spectral sidebands appear at positions corresponding to four frequency combinations: the meshing frequency ($f_{m}$), the sun gear fault frequency ($f_{s}/f_{s}^{r}$), the planet gear distributed fault frequency ($f_{p}^{r}$), and the planet carrier rotational frequency ($f_{c}$).

As shown in Figure 18 and Figure 19, the spectral characteristics of the sun gear differ under normal, tooth breakage, and wear conditions. Tooth breakage, as a local fault, results in numerous sidebands appearing near the meshing frequency and its harmonics, with additional sidebands and increased amplitudes compared with the normal state. This phenomenon is caused by unbalanced impact responses during the gear meshing process. In contrast, wear is classified as a distributed fault, in which more sidebands appear around the meshing frequency and related fault frequencies, with an overall increase in amplitude, reflecting the modulation effects induced by the gradual degradation of the tooth surface. By comparing the simulated and theoretical angular velocities of the gears, the results show minimal differences, validating the accuracy of the simulation model. The time-domain and frequency-domain characteristics of the planet gear contact forces under different operating conditions of the sun gear were analyzed, further confirming the correctness of the dynamic model considering time-varying path effects. Regarding the vibration characteristics of the planetary gearbox, the vibration patterns under various operating states were summarized. Compared to the contact force signals, the dominant frequency bands of the vibration signals are 3$f_{c}$, reflecting the combined modulation effect of the three planet gears. Additionally, the vibration signal spectrum contains distributed faults of the planet gears, revealing more fault information than the contact force signals.

In summary, the rigid-flexible coupled model of the planetary gearbox, which considers time-varying path effects, can effectively simulate the actual operating conditions and accurately reflect the dynamic process of gear meshing vibrations and fault characteristics. This provides reliable data support for the development of the planetary gearbox integrated morphology–performance digital twin system.

### 4.2. Establishment of the Planetary Gearbox Dynamic Performance Database

To store and manage the planetary gearbox’s dynamic performance data, a database must be established. It systematically records data such as contact forces and vibration characteristics under different operating conditions, facilitating access by the integrated morphology–performance system. MySQL is a widely used relational database for managing structured data, suitable for storing equipment parameters and fault information of planetary gear systems. In the context of planetary gearbox management, basic equipment attributes can be normalized and managed through data tables, with primary key constraints ensuring data uniqueness. For high-frequency vibration signals with tens of thousands of sampling points per second, a time-series database is employed, establishing cross-database data associations via device ID and timestamp. This architecture retains SQL’s support for complex queries while avoiding performance bottlenecks associated with a single database handling massive time-series data. It provides complete data support for subsequent fault prediction models and reduces redundant storage costs for historical fault data.

**Figure 20:**
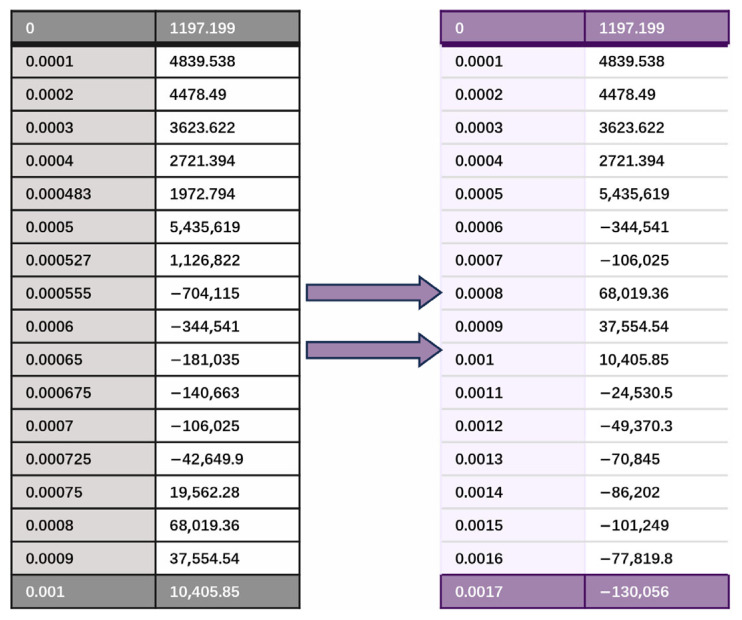
Data Processing.

During the dynamic simulation of the planetary gearbox, Adams uses a variable-step integrator based on error-controlled adaptive step size, where the time step dynamically adjusts according to the system state to ensure numerical accuracy and efficiency. As a result, the output data points are not strictly evenly spaced, which reduces the computational efficiency of the shape–performance integrated system. While this feature improves calculation accuracy, it can affect subsequent uniform sampling and database storage. To address this, a sampling frequency of 10,000 Hz is set, and during data processing only the preset sampling points are retained to ensure temporal consistency, as shown in Figure 20.

The dynamic performance database needs to support multi-dimensional data management, as well as data import and export functionalities. In this study, the PyMySQL library is used to implement standardized management of vibration signals (time-domain and frequency-domain features), dynamic contact force data, and operational status labels (normal, tooth breakage, and wear). The database is designed with standardized data interfaces, supporting batch import of field-acquired experimental data and enabling the export of complete feature datasets of the planetary gearbox based on query conditions, including equipment parameters and fault labels. This database not only meets the requirements of the shape–performance integrated system for data storage, access, and sharing but also provides data support for system expansion and model training.

Table 5 illustrates the storage format of the dynamic performance data. The first column of the table records the sampling time; the second column lists the contact force values between the sun gear and planet gears at the corresponding sampling times; the third column contains the contact force values between the planet gears and the internal ring gear; and the fourth column records the overall vibration simulation signals of the planetary gearbox. The database stores data for three operating states of the sun gear: normal, tooth breakage, and wear.

### 4.3. Establishment of the Morphology–Performance Integrated System Drive Model

The experimental signals obtained from the integrated power transmission fault diagnosis test bench are used as the driving source for the morphology–performance integrated system, with the fault diagnosis results serving as the input for the structural performance data. Simultaneously, the retrieval of data from the dynamic performance database also depends on the fault diagnosis results. Therefore, a fault diagnosis model based on experimental signals must be established to accurately identify the operating state of the planetary gearbox.

(1) Experimental Data Acquisition

**Figure 21:**
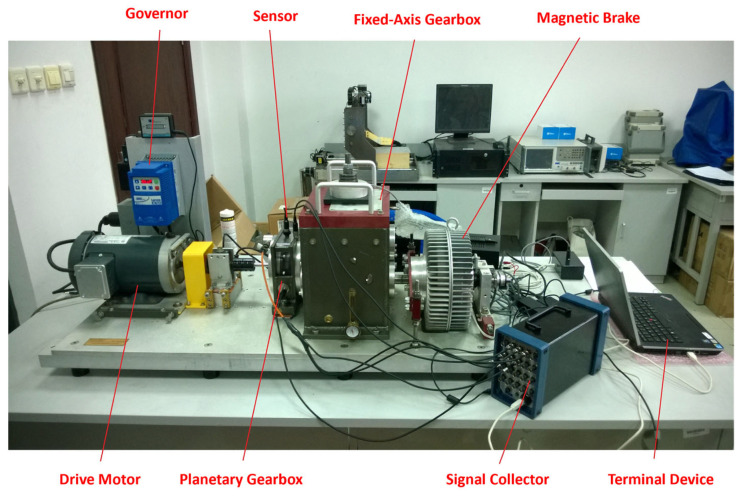
Integrated Power Transmission Fault Diagnosis Test Bench.

Experimental data were collected using the integrated power transmission fault diagnosis test bench, which consists of a drive motor, planetary gearbox, fixed-axis gearbox, and magnetic brake, as shown in Figure 21. The sampling frequency was 12,800 Hz with a total duration of 60 s. The motor operated at a constant speed of 40 Hz with a load of 10 N·m. A triaxial accelerometer was used to capture the vibration signals under three different conditions: normal, sun gear tooth breakage, and sun gear wear. The y-direction signal was selected for analysis.

(2) Model Development

**Figure 22:**
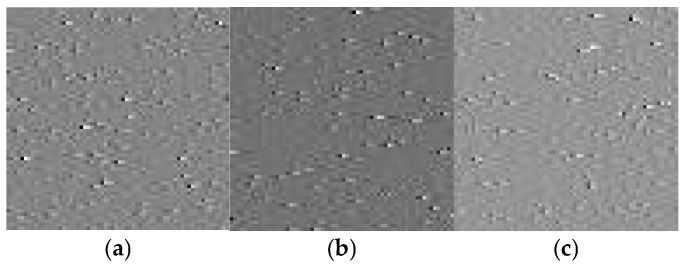
Grayscale image. (**a**) Normal Condition. (**b**) Tooth Breakage Condition. (**c**) Wear Condition.

**Figure 23:**
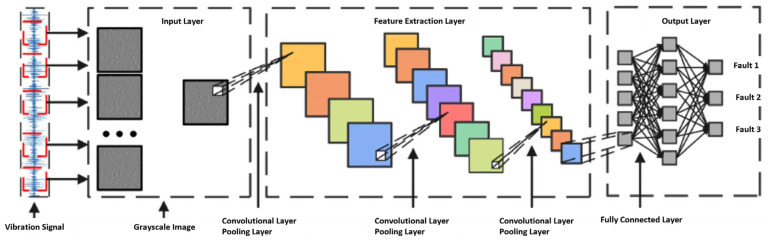
CNN model architecture diagram.

To drive the planetary gearbox shape–performance integrated system, a deep learning algorithm—Convolutional Neural Network (CNN)—is selected for model training. Leveraging its powerful feature extraction capability, CNN provides accurate operational state assessments for the system. The model converts one-dimensional experimental signals into grayscale images using a sliding window approach, as illustrated in Figure 22. These grayscale images are then fed into the constructed CNN model to extract critical fault features. To enhance fault recognition capabilities, the model utilizes three sets of convolutional and pooling layers. The convolutional layers aim to extract deep features, while the pooling layers reduce the feature map size by half, enabling thorough extraction of key characteristics in the images. Finally, the fully connected layer maps neurons to output the fault category. The structure and parameters of the model are shown in Figure 23 and Table 6, respectively.

(3) Result Analysis

Based on the model parameters, 132,032 experimental data points were selected for each condition: normal state, sun gear tooth breakage, and sun gear wear. Grayscale images were generated using overlapping sliding window sampling with a window size of 4096 and a stride of 64. Each fault mode produced 2000 samples, totaling 6000 samples. The generated grayscale images were randomly divided into training and testing sets at an 8:2 ratio. The experimental parameters were set as follows: training batch size of 32, testing batch size of 8, learning rate of 1 × 10^−6^, training performed using the ADAM adaptive optimizer, and the loss function chosen was cross-entropy loss.

**Figure 24:**
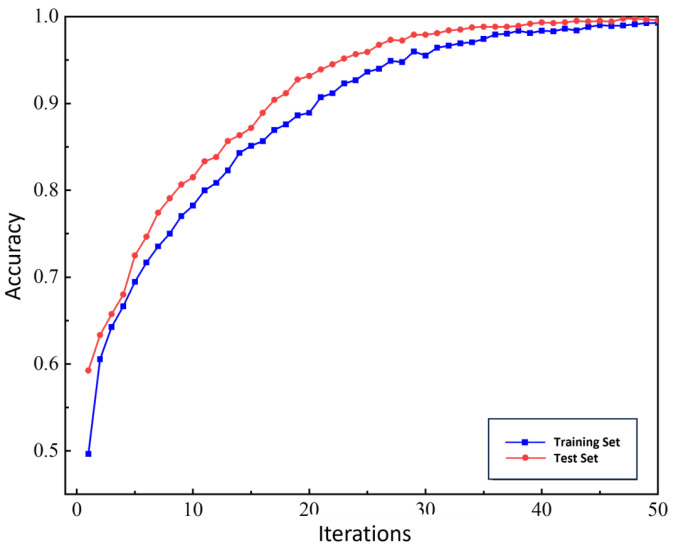
Fault Identification Accuracy Curve.

Based on the above parameters, the grayscale images were input into the CNN model for 50 iterations. The training and testing accuracy curves are shown in Figure 24, with the final recognition accuracy reaching 99%. The training process exhibited minimal fluctuations, indicating that the model can accurately distinguish experimental signals from planetary gearboxes under different fault conditions.

**Figure 25:**
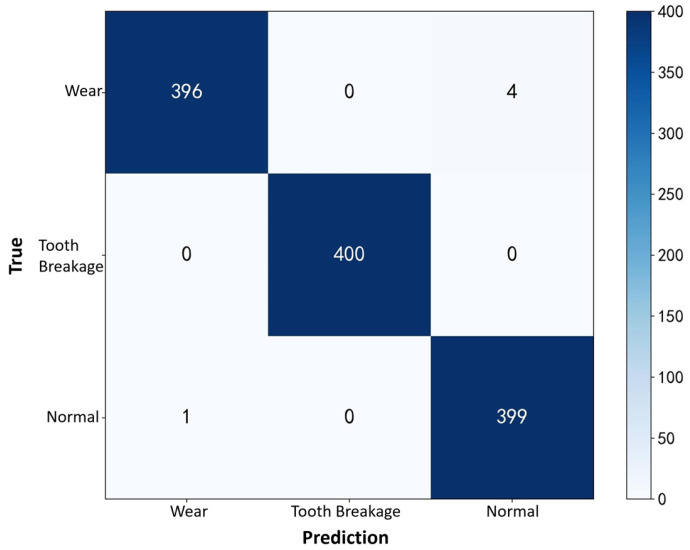
Confusion Matrix of the CNN Model.

To further demonstrate the CNN’s ability to identify fault types in the planetary gearbox, a confusion matrix was plotted to clearly display the classification counts, as shown in Figure 25. Among the 400 samples in the test set, the recognition rate for sun gear tooth breakage reached 100%, while the recognition rates for the normal state and wear faults both reached 99%. This slight misclassification is attributed to the low degree of sun gear wear, which causes its features to closely resemble those of normal samples. Overall, the constructed CNN model exhibits high fault recognition accuracy for experimental signals across different operating conditions.

During the operation of the planetary gearbox, gears are prone to failure under prolonged high-speed rotation and sustained load conditions due to various factors, such as instantaneous overload, material fatigue, and intrusion of foreign objects. These factors can lead to typical faults, including tooth breakage, wear, and pitting. Among them, tooth breakage and wear are more prevalent; therefore, this study selects sun gear tooth breakage and tooth surface wear as the fault modeling targets.

**Figure 26:**
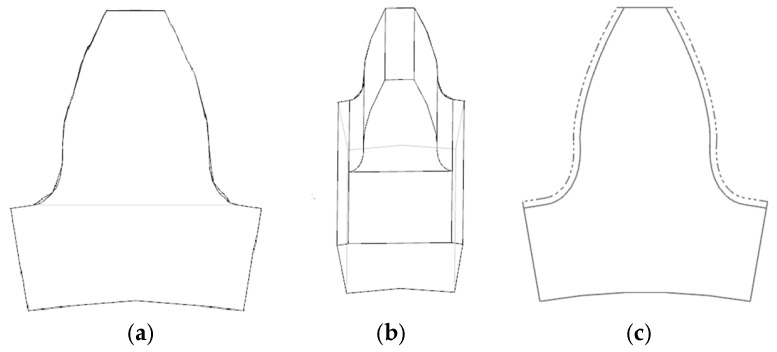
Sun Gear Models under Different Operating Conditions. (**a**) Normal. (**b**) Tooth Breakage. (**c**) Wear.

The tooth breakage fault is characterized by a crack appearing at a 10 mm thickness of the sun gear tooth, extending approximately 5 mm along the tooth direction, showing typical bending fatigue fracture features. The tooth surface wear fault manifests as gradual material removal caused by contact stress and friction. The material loss due to fatigue wear is represented by defects of 0.1 mm on both sides along the tooth width at the sun gear pitch circle thickness (1.57 mm). Figure 26 presents comparative models of the sun gear in normal, tooth breakage, and wear conditions.

Based on the quantitative description of fault size and severity above, it can be seen that the selected faults represent relatively typical and pronounced failure cases. These settings ensure that the digital twin model can fully reflect fault characteristics during the preliminary validation phase, thereby allowing assessment of its feasibility for fault detection and performance prediction. In subsequent studies, we will further consider more subtle fault scenarios, such as early-stage cracks and minor wear, to comprehensively evaluate the model’s generalizability and robustness under various complex operating conditions.

## 5. Establishment of the Planetary Gearbox Form-Performance Integrated System

### 5.1. Implementation of the Planetary Gearbox Digital Twin System

The development of digital twin systems primarily involves three approaches: low-level development, computer language programming, and specialized software. Low-level development uses tools such as DirectX and Trace32, but its high demands on hardware and programmer expertise make development challenging. Computer language programming employs tools like Java3D and JetBrains, providing functional modules aligned with user operation habits. Specialized software development relies on platforms such as Unity3D and Unix, improving development efficiency by reducing the amount of programming required. In comparison, development based on specialized software meets system requirements while shortening development time. A comparison of relevant professional software in terms of visual quality, interactivity, compatibility, and cross-platform capability is provided in Table 7.

Unity3D demonstrates excellent performance in various aspects, with its strong compatibility and cross-platform capabilities being particularly notable. Considering these factors, Unity3D was selected as the platform for the planetary gearbox shape–performance integrated system in this study.

The planetary gearbox shape–performance integrated system is divided into the system platform and the backend data module, and is developed on the Windows platform. The backend data module primarily includes the structural performance module, the system driving model (fault diagnosis model), the dynamic performance module, and the data communication server. The structural performance module and the system driving model are implemented in Python, utilizing the SciPy and Torch libraries, respectively. The dynamic performance module stores data in a MySQL database, while the communication server employs Socket network communication. The planetary gearbox shape–performance integrated system platform is developed in Python, with the interactive interface built using Qt Designer. The development toolkits used are listed in Table 8:

### 5.2. System Data Backend Development

The backend data is divided into structural performance surrogate models, system driving models, dynamic performance databases, and data communication. The core of the structural performance surrogate model consists of an RBF-trained structural performance surrogate model integrated with the dynamic performance data stored in a MySQL database. The structural performance surrogate model processes data by placing it into lists and augmenting these lists with RBF interpolation functions, then encapsulates the model into *.pkl files for convenient calling. The system driving model is constructed based on a CNN algorithm. The MySQL database stores dynamic performance data according to the operating states.

**Figure 27:**
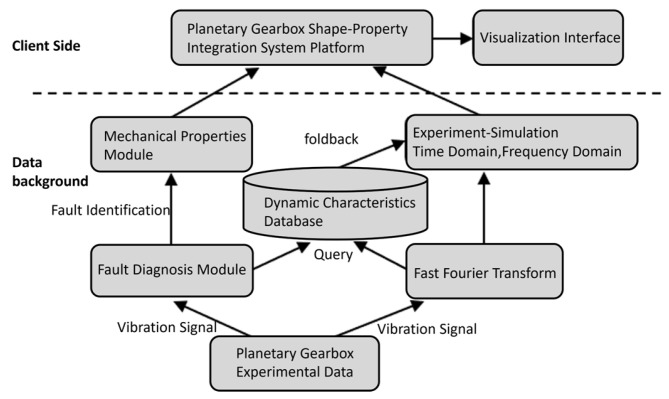
Operation Process of the Shape-Performance Integrated System.

After completing the system’s embedded development, the backend data operation flow is as follows: The backend receives vibration data from the integrated drivetrain fault diagnosis test rig during its operation. Part of the data is fed into the fault diagnosis module for fault identification, while another part is transformed into frequency-domain features via Fast Fourier Transform (FFT). Once the fault diagnosis module identifies the fault type, this information is passed to the structural performance module. The structural performance results are then transmitted to the shape-performance integrated system platform through Socket network communication. Notably, the fault diagnosis module and structural performance module operate sequentially. Experimental data undergo time-to-frequency domain conversion via FFT, and the system driving model’s recognition results query the database to retrieve corresponding dynamic performance data, which is then delivered to the planetary gearbox shape-performance integrated platform. The operational flow is illustrated in Figure 27.

**Figure 28:**
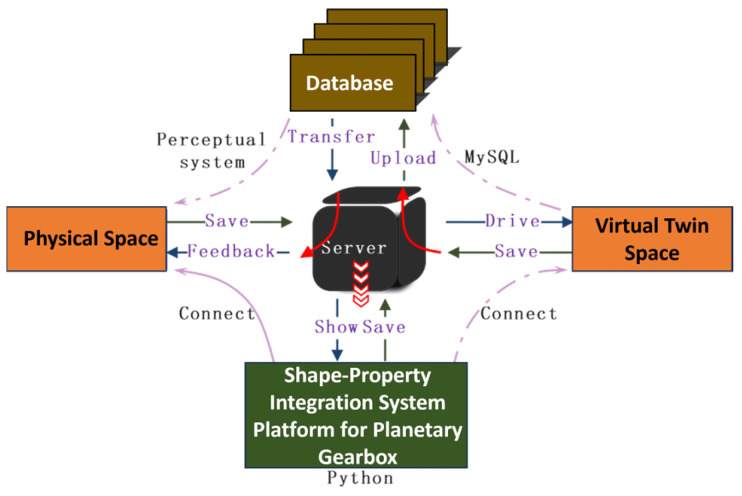
Data Interaction Flow of the Shape–Performance Integrated Planetary Gearbox Monitoring System.

The normal operation of the shape-performance integrated system requires data interaction among its modules. Figure 28 illustrates the data interaction process of the planetary gearbox shape-performance integrated system. Socket communication uses the TCP/IP protocol to input experimental platform data into the server. This experimental data drives the virtual twin space—composed of the structural performance proxy model and fault diagnosis model—for fault diagnosis and structural performance prediction, with the visualization module displaying the prediction results. The virtual twin space uploads the results to the database via Socket communication and calls the planetary gearbox dynamic performance data to the server. Finally, the server presents the results on the system user interface.

### 5.3. Integrated Geometry-Performance System and Functional Modules

**Figure 29:**
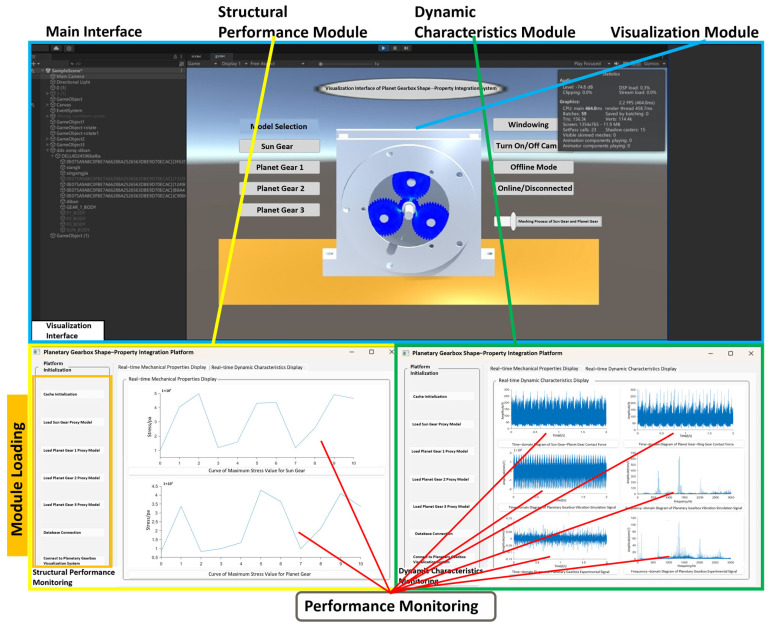
Monitoring and Operation Interface of the Planetary Gearbox Shape–Performance Integrated System.

The planetary gearbox integrated geometry-performance system is jointly developed using Unity3D and Python. All visualization modules are implemented on the Unity client, while backend data interaction runs on the Python client. Figure 29 shows the system interface, which includes the upper part as the visualization interface and the lower part as the integrated geometry-performance system platform interface. Based on the system architecture, through virtual-physical mapping and data interaction, combined with integrated functional modules, the planetary gearbox integrated geometry-performance system has been successfully established.

Based on experimental data from the comprehensive power transmission fault diagnosis test bench, the integrated geometry-performance system was driven and analyzed, with results shown in Figure 29. The overall system architecture mainly consists of three parts: the structural performance module, the dynamic performance module, and the visualization module. These correspond, respectively, to structural performance prediction, dynamic performance display, and interactive visualization of results. The structural performance module monitors the maximum stress values of the planetary gear box under different operating states (such as normal, tooth breakage, and wear). It uses the surrogate model to predict stresses and extract data, reflecting the stress variation patterns of the sun gear in various operating conditions. The dynamic performance module retrieves and analyzes contact forces and vibration simulation signals in real time. It covers contact force variations between the sun gear and planet gears, between the planet gears and internal ring gear, and vibration responses of the planetary gearbox from both simulation and experimental signals. This module provides fault diagnosis capability in both time and frequency domains. The visualization module presents the structural performance visually, showing the overall stress distribution of the sun gear, visual feedback under fault conditions, and early warning prompts for faults, enhancing the system’s intuitive interactivity.

(1) Structural Performance Function

**Figure 30:**
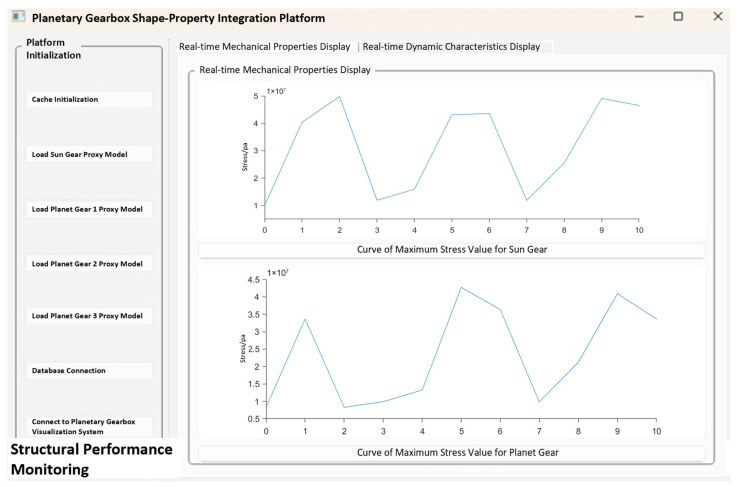
Operational Status of the Structural Performance Module in the Shape–Performance Integrated System.

Figure 30 shows the maximum stress prediction results of the sun gear and planet gears in the structural performance module during the operation of the planetary gearbox. After receiving the experimental signals, the system uses the driving model to identify the current operating state and uploads the identification results to the structural performance module. According to the fault type, the structural performance module automatically matches and calls the corresponding surrogate model to achieve real-time prediction and updating of the sun gear’s structural performance under the current operating condition of the planetary gearbox. During the prediction process, users can select any time point within the meshing process via a slider to dynamically observe the maximum stress variation trend of a single sun gear tooth at different meshing positions, enabling dynamic perception of the structural response.

By comparing the predicted maximum stress of the sun gear throughout the meshing process, the structural performance module’s prediction accuracy can be validated. This also allows assessment of the surrogate model’s adaptability under different operating conditions. Considering that the planet gears primarily serve auxiliary functions in the system and have a relatively minor impact on overall structural performance, their prediction accuracy is not verified within this module.

**Figure 31:**
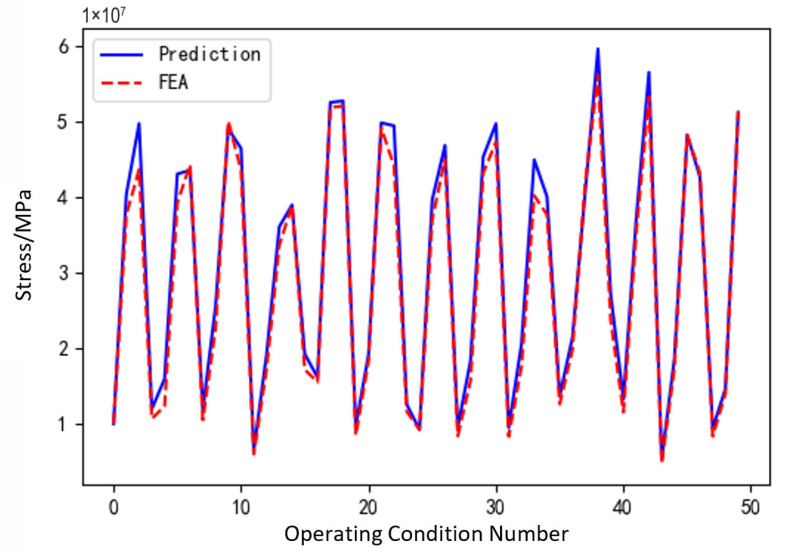
Comparison between predicted and actual values of the sun gear under normal condition.

**Figure 32:**
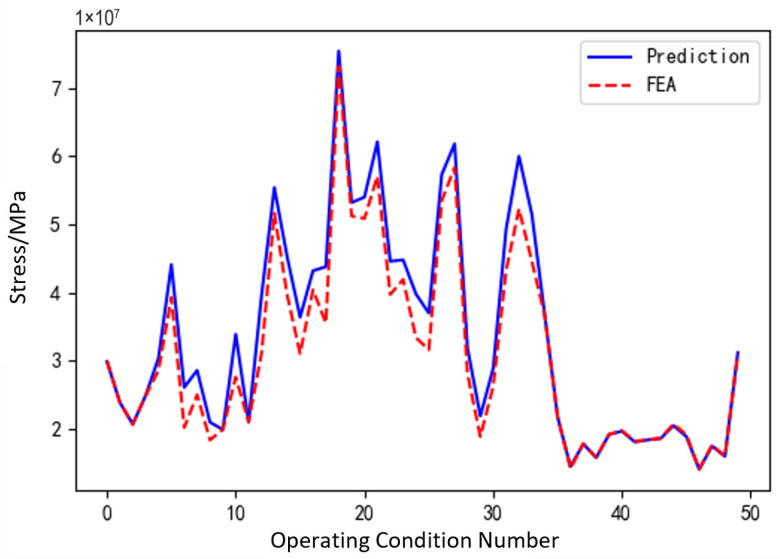
Comparison between predicted and actual values of the sun gear under tooth breakage condition.

**Figure 33:**
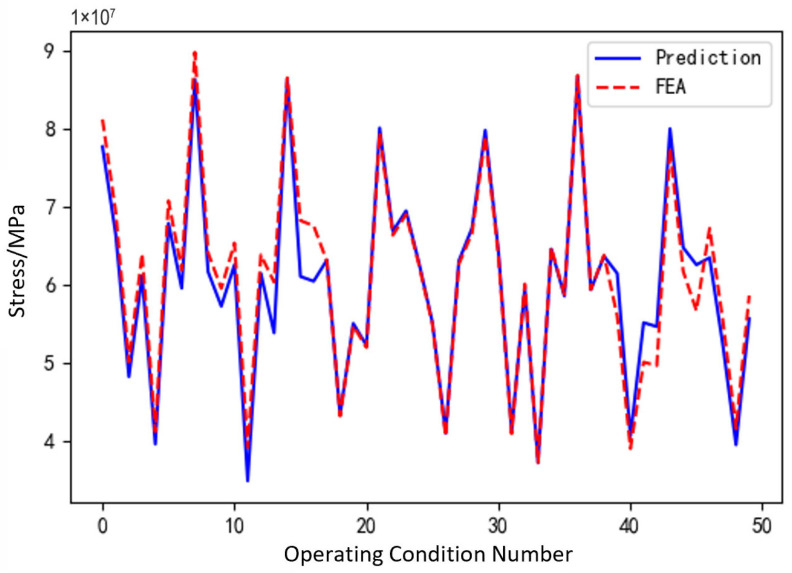
Comparison of predicted and actual values of the sun gear under wear condition.

Figure 31, Figure 32 and Figure 33, respectively, show the comparison curves of the maximum stress prediction values and simulation values of the sun gear under normal, tooth breakage, and wear conditions. In the figures, the red dashed line represents the simulation values, and the blue solid line represents the prediction values. The horizontal axis corresponds to the entire meshing process of a single sun gear tooth, divided evenly into fifty operating conditions. When the sun gear is in a normal state, the maximum stress curve exhibits relatively stable periodic fluctuations. The prediction values highly coincide with the finite element analysis results, with a maximum stress value of 56.25 MPa, demonstrating that the surrogate model has good fitting accuracy under normal conditions. When the sun gear has a tooth breakage, the stress curve shows obvious changes, with abnormal stress increases occurring under some conditions. The predicted maximum stress value is 73.13 MPa. Although some peak positions slightly deviate from the FEA results, the surrogate model still fits the overall trend of maximum stress well. In the case of sun gear wear, the stress curve shows larger amplitude fluctuations, and the overall stress level rises, with some peaks exceeding the normal state. The maximum stress value is 86.83 MPa. The prediction error of the maximum stress value under the wear condition is the largest at 3%, which only occurs in individual conditions and has little impact on the performance evaluation of the entire meshing process of the sun gear tooth. By predicting the maximum stress value throughout the entire meshing process of a single sun gear tooth under different operating conditions of the planetary gearbox, the accuracy of the structural performance module is verified.

(2) Dynamic Performance Function

**Figure 34:**
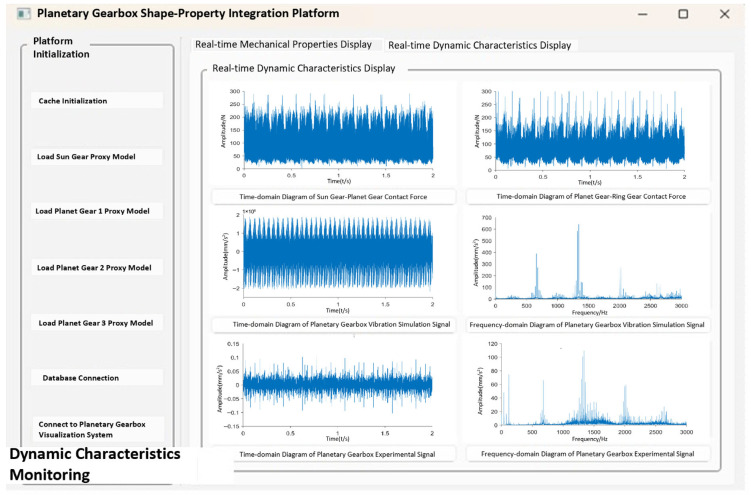
Invisible Monitoring Parameter Curve Diagram.

Figure 34 shows the operation results of the dynamic performance module. After the system completes the identification of the operating state, the dynamic performance module calls the simulation data corresponding to the operating state from the database in real time, while simultaneously receiving and processing some vibration signals from the experimental platform. Inside the module, experimental and simulation signals from the database are uploaded to the planetary gearbox integrated shape–performance system platform. These signals support evaluation of dynamic response characteristics and display of dynamic performance under the current operating state.

The validation of the dynamic performance module is carried out by selecting vibration simulation signals corresponding to the experimental signals. Since the morphology–performance integrated system employs FFT for time-frequency domain conversion, resulting data segments under different operating states are extracted from the system and transformed using FFT for analysis.

**Figure 35:**
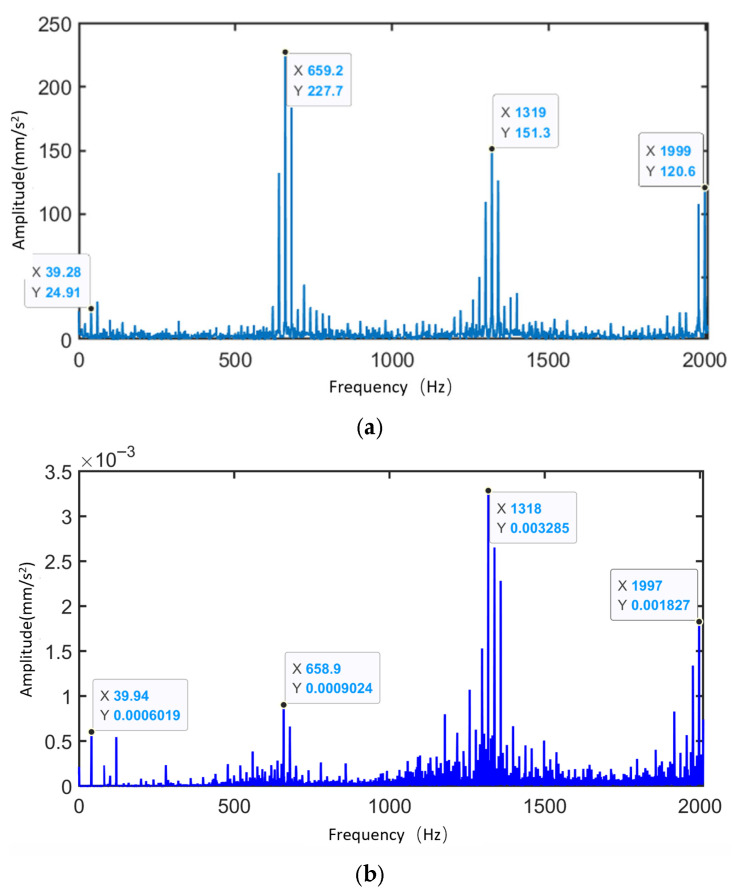
Data spectrum analysis of shape and property integration system under normal state of solar wheel. (**a**) Simulation Signal Spectrum Diagram. (**b**) Experimental Signal Spectrum Diagram.

Figure 35 presents the frequency spectra of the vibration simulation signal and experimental signal of the planetary gearbox under the normal condition of the sun gear. The figure highlights the dominant sidebands near the sun gear rotational frequency, the meshing frequency, and their harmonics. Table 9 compares the peak frequencies observed in the experiment, simulation, and theoretical calculations. From the frequency distribution, the main peaks in the experimental signal appear at 39.94 Hz, 658.9 Hz, 1318 Hz, and 1997 Hz, while the simulation signal peaks are observed at 39.28 Hz, 659.2 Hz, 1319 Hz, and 1999 Hz. The two sets of data show high consistency in their dominant frequency components, with minimal frequency deviation. The maximum error between simulation and experimental signals is 1.68%, which is less than 2%, aligning well with the theoretical values at the sun gear rotational frequency ($f_{a}$). This demonstrates the system’s capability to accurately reflect the dynamic behavior of the physical gearbox.

**Figure 36:**
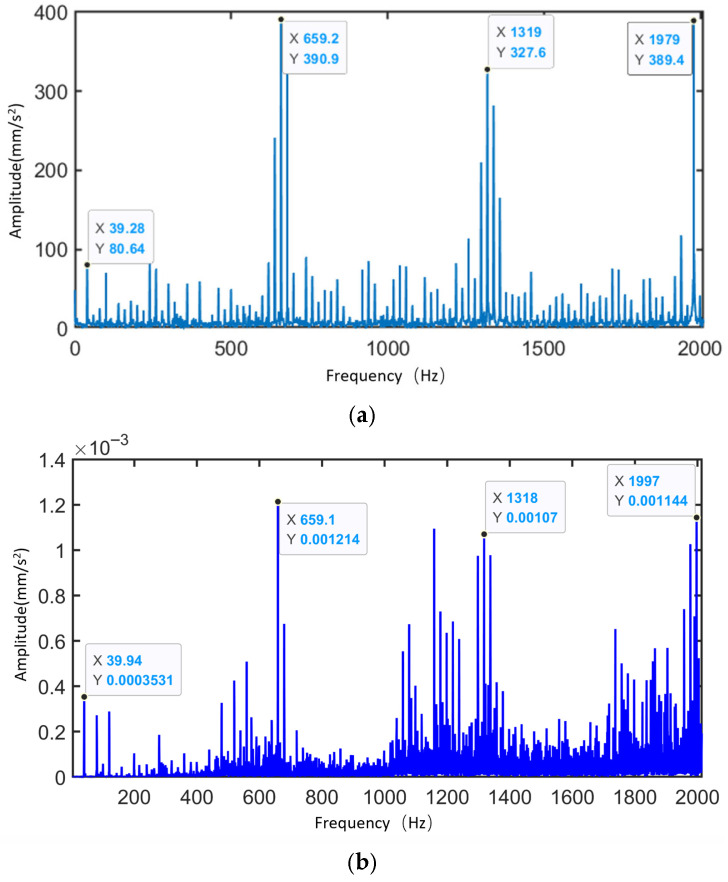
Data spectrum analysis of the shape and property integration system under the condition of broken teeth of the sun gear. (**a**) Simulation Signal Spectrum Diagram. (**b**) Experimental Signal Spectrum Diagram.

Figure 36 shows the vibration spectrum of the planetary gearbox under the sun gear tooth breakage condition, including both simulation and experimental signals. The figure marks the sun gear rotational frequency, mesh frequency, and the sidebands with the largest amplitudes near the harmonic frequencies. Table 10 compares the frequencies of the largest sidebands from experimental, simulation, and theoretical results. The maximum error between the main sideband frequencies in the experimental and simulation signals is 1.68%, occurring at the sun gear rotational frequency. At the triple mesh frequency in the simulation signal, the position of the spectral peak changes, and the largest sideband is modulated by the planet carrier rotational frequency (the planet gear orbital frequency), with the largest sideband at $3f_{m}-3f_{c}$.

**Figure 37:**
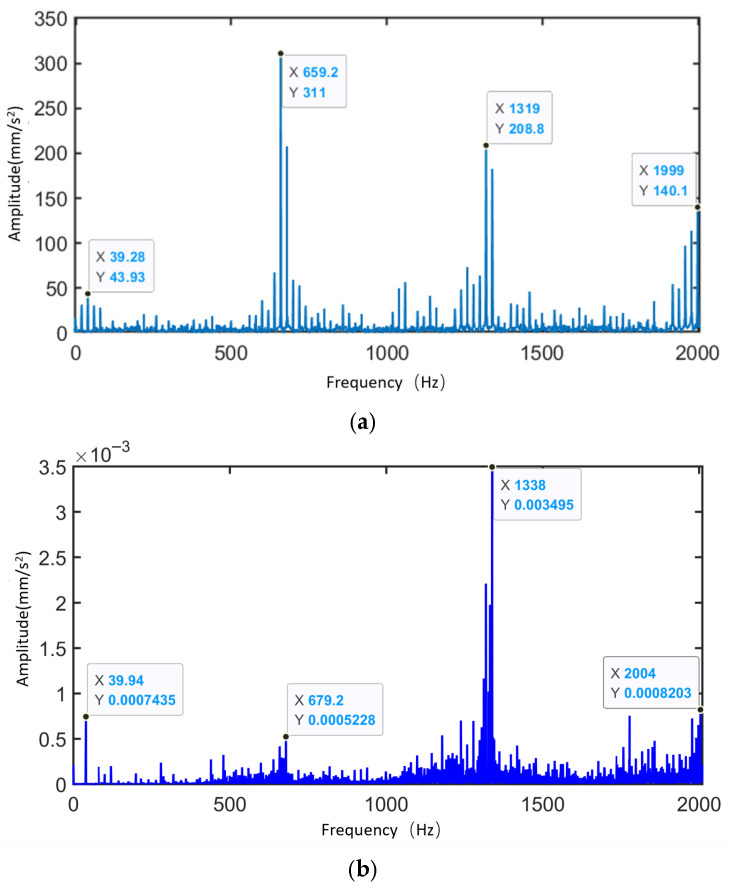
Data spectrum analysis of shape and property integration system under the condition of sun wheel wear. (**a**) Simulation Signal Spectrum Diagram. (**b**) Experimental Signal Spectrum Diagram.

Figure 37 shows the vibration spectrum diagrams of the planetary gearbox under the sun gear wear condition, including both simulation and experimental signals. The figure marks the sun gear rotational frequency, mesh frequency, and the largest sideband amplitudes near their harmonics. Table 11 compares the maximum sideband frequencies from experiments, simulations, and theoretical calculations. The maximum deviation between the experimental and simulation sideband frequencies is 1.68%, occurring at the sun gear rotational frequency. Due to the presence of wear faults, the largest sidebands near the mesh frequency and its harmonics in both experimental and simulation signals are modulated by the planetary carrier rotational frequency.

Zoom in on the experimental signal near the fundamental mesh frequency to further analyze the spectral patterns of the experimental signal.

**Figure 38:**
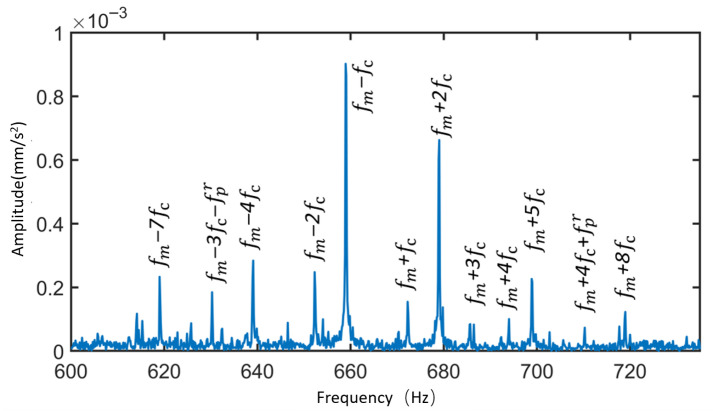
Local amplification of normal state frequency domain.

Figure 38 shows the time-domain and frequency-domain diagrams of vibration signals collected by the accelerometer under normal conditions. The analysis results are as follows:

Under the normal condition of the sun gear, the modulation effect caused by the revolution of the planetary gears dominates, resulting in the appearance of primary sidebands in the frequency spectrum, including $f_{m}-f_{c}\pm \mathrm{Na}f_{c}$. Due to inevitable machining and assembly errors in the planetary gear system, there are slight differences in the impact forces when the three planetary gears mesh with the sun gear, leading to the emergence of additional sidebands near the primary ones, such as $f_{m}-f_{c}\pm af_{c}/2af_{c}$.

Vibration characteristic analysis indicates that the experimental signals of the planetary gearbox under normal conditions exhibit an overall pattern consistent with the simulation results. Due to manufacturing and installation errors of the actual planetary gears, additional sideband components appear between the dominant sidebands in the experimental signals. Furthermore, the experimental signals are also modulated by the distributed faults of the planetary gears, such as $f_{p}^{r}$.

**Figure 39:**
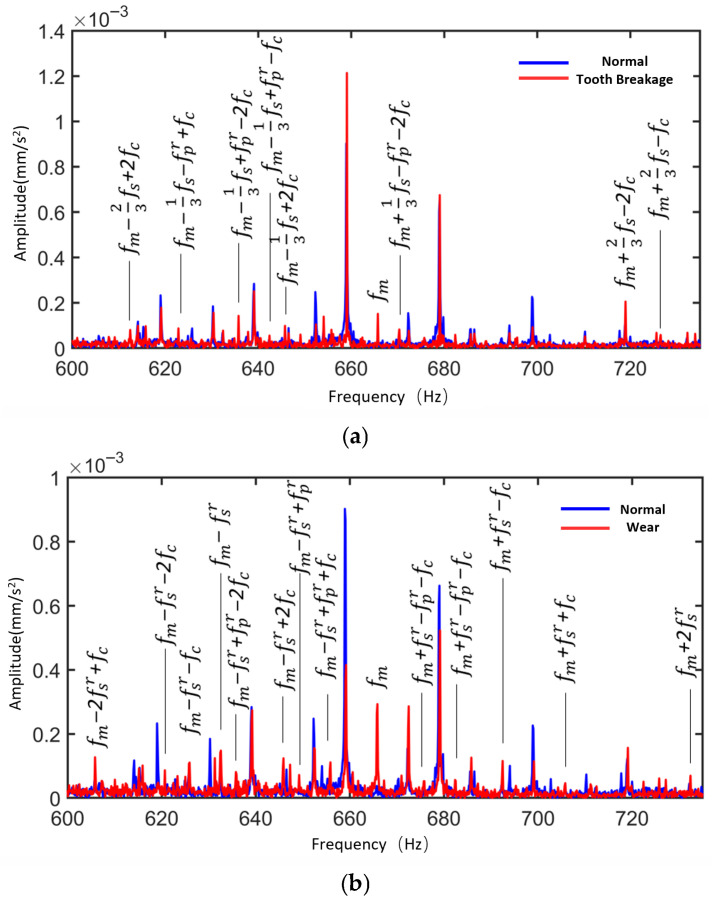
The spectral distribution diagram at the first harmonic of the meshing frequency under sun gear fault condition. (**a**) The locally enlarged frequency-domain comparison diagram of broken tooth versus normal condition. (**b**) The locally enlarged frequency-domain comparison diagram of wear versus normal condition.

Based on Figure 39, it can be concluded that:

The local fault experimental signals contain $f_{m}\pm \frac{a}{N}f_{s}\pm bf_{c}\pm df_{p}^{r}$ sidebands, and the sideband amplitudes related to the sun gear local fault frequency are significantly enhanced. In contrast, the distributed fault experimental signals exhibit characteristics including the $f_{m}\pm af_{s}^{r}\pm bf_{p}^{r}\pm df_{c}$ sidebands. Vibration characteristic analysis indicates that the simulated signals and experimental signals generally follow the same patterns. (Note: *N* = 3, a, b, d = 0, 1, 2, …).

In summary, by retrieving simulation signals from the database and conducting frequency-domain comparative analysis with experimental signals, the consistency between them in terms of main frequency components and spectral distribution patterns was verified. The results demonstrate that the dynamic model can effectively simulate the actual dynamic performance of the planetary gearbox. Additionally, the above analysis validates the accuracy of the dynamic performance module.

(2) Visualization function

**Figure 40:**
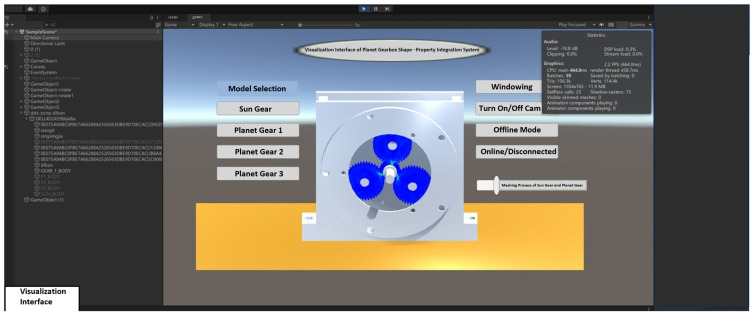
Interactive Operation Diagram of the Shape–Performance Integrated Visualization Module.

Figure 40 shows the results of the visualization module in operation. It demonstrates the real-time interaction and dynamic response of the structural performance model during system operation. The modular display allows observation of the coordination in the meshing motion between gears.

**Figure 41:**
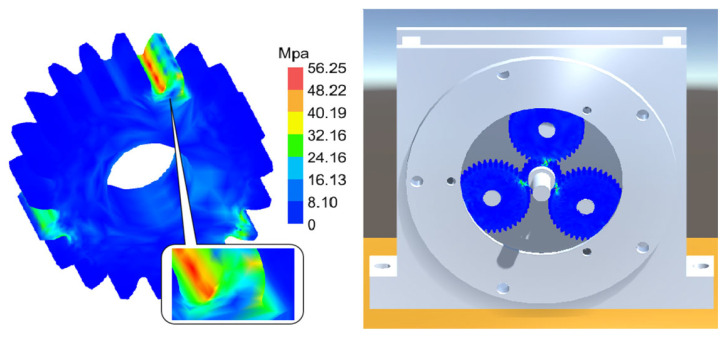
Visual stress cloud diagram of solar wheel in normal state.

**Figure 42:**
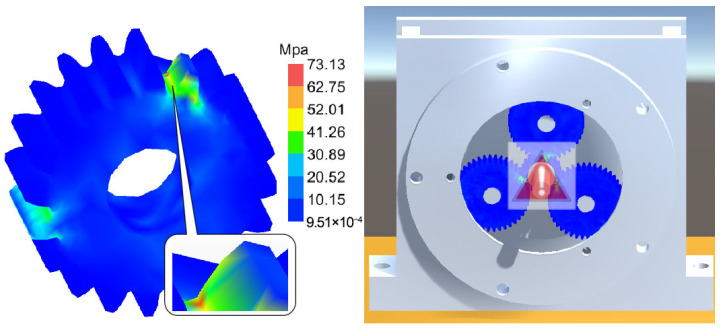
Visual stress cloud diagram of broken tooth state of sun gear.

**Figure 43:**
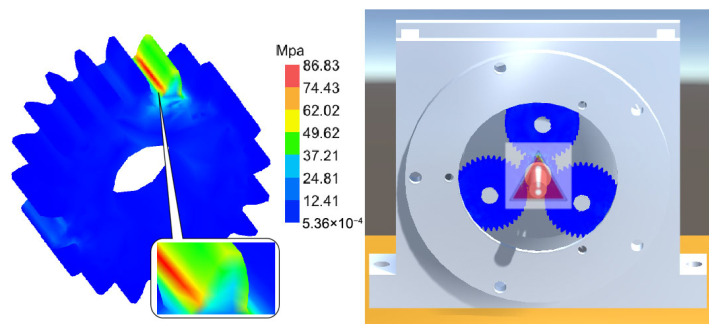
Visual stress cloud diagram of sun wheel wear state.

Figure 41, Figure 42 and Figure 43 present stress visualization cloud maps of the planetary gearbox under different operating conditions. The left side shows a localized enlarged view of the sun gear, where stress values in each region can be referenced through the corresponding color threshold legend. In the normal state of the planetary gearbox, the maximum stress on the sun gear is evenly distributed at the tooth root. Under the broken tooth condition, the maximum stress concentrates at the tooth root fracture area. In the wear condition, contact at the tooth tip induces stress concentration at the tooth root; overall stress distribution remains relatively uniform, but the stress levels increase globally. By combining the cloud map colors with the threshold legend, the stress distribution characteristics in different gear regions are intuitively reflected, aiding the assessment of the planetary gearbox’s operating condition and enabling visualization of its operational state and stress distribution. Comparison of multi-fault stress fields in the planetary gearbox indicates that the visualized stress distribution maps closely match the simulation results, validating the accuracy of the visualization module.

Based on experimental data-driven analysis, the operational results of the structural performance module, dynamic performance module, and visualization module of the planetary gearbox integrated digital twin system are verified. These results confirm that the system can achieve physical entity operational state recognition, performance prediction, and analysis.

## 6. Conclusions

(1) In response to the requirements for operational state recognition and performance prediction of the planetary gearbox, a system solution integrating both “morphology” and “performance” was developed. The design objectives and development process of the system were planned, a hierarchical architecture for the planetary gearbox was proposed, and the functional modules were reasonably partitioned. Key technologies necessary for system establishment were also elucidated.

(2) By integrating the finite element method with neural network algorithms, the structural performance model was established. In constructing the surrogate model, a radial basis function neural network algorithm based on the Gaussian kernel demonstrated excellent fitting accuracy. Model evaluation results showed that the surrogate model’s coefficient of determination (R^2^) exceeded 0.9, indicating strong predictive capability. Among the conditions, the model error was largest under the sun gear wear state, with a root mean square error (RMSE) of 2.1 MPa and a mean absolute error (MAE) of 0.9 MPa. However, relative to the maximum stress level of 86.278 MPa under the wear condition, this error remains within an acceptable range, meeting the precision requirements of the integrated morphology–performance system.

(3) For the dynamic performance analysis of the planetary gearbox, the angular velocity, contact forces, and vibration characteristics were examined. Meanwhile, a driving model for the integrated morphology–performance system was developed based on deep learning algorithms, utilizing experimental data as input to drive the system’s operation. The results demonstrate that the integrated morphology–performance driving model achieves an identification accuracy exceeding 99% for the experimental signals.

(4) The integrated morphology–performance system for the planetary gearbox was established by integrating and developing rapid operational state recognition and performance prediction functionalities. A system interactive interface was implemented, and the system was validated using experimental signals from a comprehensive experimental platform for power transmission fault diagnosis. The results indicate that the structural performance module, dynamic performance module, and visualization module of the system all meet the expected objectives.

The validation in this study is primarily based on a comparison between experimental signals and simulation results at typical time points, aiming to assess the basic effectiveness of the proposed model in gear defect identification. While this approach demonstrates the feasibility of the model, it does not yet cover system validation of the gearbox under different degradation stages, such as early crack initiation, progressive wear expansion, and severe tooth breakage.

On the other hand, the surrogate model uses operating condition data at different meshing angles (frames) as input and outputs the predicted stress values for the corresponding vertices. For each vertex, the model establishes a numerical expression based on the available condition data, with its applicability mainly focused on the meshing process of a specific tooth of the sun gear. When applied beyond this range, the model’s accuracy may be affected, potentially resulting in underfitting or overfitting. It should be noted that the stress data in this study are primarily derived from finite element simulations, i.e., numerical simulations of the gear transmission process. Therefore, the fidelity of the finite element model directly affects the reliability of the digital twin model’s predictions. In future research, we plan to further expand the data range and incorporate richer operating condition information to enhance the model’s generalizability and robustness.

## Figures and Tables

**Table 1:** Comparison of Surrogate Model Accuracy under Normal Sun Gear Conditions Using Different Algorithms.

Component	Surrogate Model Type	R^2^	RMSE	MAE
Sun Gear	RBF	0.9141	1.4 × 10^6^	7.6 × 10^5^
	GPR	0.8944	1.6 × 10^6^	7.1 × 10^5^
	RF	0.7157	2.6 × 10^6^	1.3 × 10^6^

**Table 2:** Evaluation Results of the Surrogate Model under Different Operating Conditions of the Sun Gear.

Component	Fault Type	R^2^	RMSE	MAE
Sun Gear	Normal	0.9141	1.4 × 10^6^	7.6 × 10^5^
	Tooth Breakage	0.9059	8.3 × 10^5^	3.7 × 10^5^
	Wear	0.9011	2.1 × 10^6^	9.1 × 10^5^

**Table 3:** Rotational Speeds of Components under 10 N·m Torque.

Average Rotational Speed	Theoretical Value (°/s)	Simulated Value (°/s)	Relative Error (%)
Sun Gear	14,400	14,400	0
Planet Gear	3600	3603.12	0.087
Planet Carrier	2400	2402.37	0.099

**Table 4:** Frequency Notation Information.

Symbol	Name	Theoretical Value	Symbol	Name	Theoretical Value
$f_{m}$	Meshing Frequency	666.67	$f_{s}$	Local Fault of Sun Gear	100
$f_{c}$	Planet Carrier Rotational Frequency	6.67	$f_{s}^{r}$	Distributed Fault of Sun Gear	33.33
$f_{a}$	Sun Gear Rotational Frequency	40	$f_{p}^{r}$	Distributed Fault of Planet Gear	16.67

**Table 5:** Example Table of the Dynamic Performance Database Section.

Time	Sun-Planet	Planet-Ring Gear	Vibrate
0	0	0	−11.97199
0.0001	5.59 × 10^−19^	1.44 × 10^−18^	−48.39536
0.0002	1.23 × 10^−18^	3.13 × 10^−18^	−44.78462
0.0003	1.92 × 10^−18^	4.82 × 10^−18^	−36.23549
0.0004	2.61 × 10^−18^	6.51 × 10^−18^	−27.21274
0.0005	341.1147	69.42069	−3304.092
0.0006	71.33642	53.75457	284.1585
0.0007	52.84328	42.9924	−443.4701
0.0008	64.1431	55.48964	−896.7224
0.0009	68.47795	59.70228	−1005.638

**Table 6:** Model parameters.

Network Architecture	Convolution Kernel	Stride	Padding	Number of Channels	Output Size
Input layer	-	-	-	-	64 × 64
Convolutional Layer 1	5 × 5	1	2	32	64 × 64
Pooling Layer 1	2 × 2	2	-	32	32 × 32
Convolutional Layer 2	5 × 5	1	2	64	32 × 32
Pooling Layer 2	5 × 5	5	-	64	16 × 16
Convolutional Layer 3	3 × 3	1	1	128	16 × 16
Pooling Layer 3	2 × 2	2	-	128	8 × 8
Fully connected layer	-	-	-	-	8192 × 256
Output layer	-	-	-	-	256 × 3

**Table 7:** Performance Comparison of Common Digital Twin Development Software.

Software	Interactivity	Picture Quality	Can It Be Cross Platform	Compatibility
Unix	strong	strong	Cannot cross platform	average
Labview	average	strong	Cannot cross platform	weak
VRP	average	average	Cannot cross platform	average
Eon	weak	strong	Cannot cross platform	strong
Unity3D	strong	strong	Cross platform capability	strong

**Table 8:** Development Toolkits.

Tool Category	Description
development platform	Windows
development language	C#, Python 3.6.8
IDE	Qt Designer, Visual Studio2019
Model development toolkit	Matplotlib 3, Numpy 1.26.4, Pandas 2.1.4, etc.
Unity3D component (v. 2023.2.1f1c2)	UnityEngine.UI, DOTween, etc.

**Table 9:** Comparison of Main Sideband Frequencies in Vibration Signals under Normal Conditions.

Sideband Frequency	Experimental Frequency/Hz	Simulated Frequency/Hz	Theoretical Frequency/Hz
$f_{a}$	39.94	39.28	40
$f_{m}-f_{c}$	658.9	659.2	660
$2f_{m}-2f_{c}$	1318	1319	1320
$3f_{m}$	1997	1999	2000

**Table 10:** Comparison of Main Sideband Frequencies in Vibration Signals under Tooth−Breakage Conditions.

Sideband Frequency	Experimental Frequency/Hz	Simulated Frequency/Hz	Theoretical Frequency/Hz
$f_{a}$	39.94	39.28	40
$f_{m}-f_{c}$	658.9	659.2	660
$2f_{m}-2f_{c}$	1318	1319	1320
$3f_{m}-3f_{c}$	-	1979	1980
$3f_{m}$	1997	-	2000

**Table 11:** Comparison of Main Sideband Frequencies in Vibration Signals under Wear Conditions.

Sideband Frequency	Experimental Frequency/Hz	Simulated Frequency/Hz	Theoretical Frequency/Hz
$f_{a}$	39.94	39.28	40
$f_{m}-f_{c}$	-	659.2	660
$f_{m}+2f_{c}$	679.2	-	680
$2f_{m}-2f_{c}$	-	1319	1320
$2f_{m}+f_{c}$	1338	-	1340
$3f_{m}$	2004	1999	2000

## Data Availability

Data is contained within the article.

## References

[1] Barari A., Tsuzuki M.S.G. (2023). Smart Manufacturing and Industry 4.0. Appl. Sci..

[2] Vijay A., Paulson N., Sadeghi F. (2018). A 3D finite element modelling of crystalline anisotropy in rolling contact fatigue. Int. J. Fatigue.

[3] Leaman F., Vicuña M.C., Clausen E. (2021). A Review of Gear Fault Diagnosis of Planetary Gearboxes Using Acoustic Emissions. Acoust. Aust..

[4] Li D., Yang Z., Yao Z. (2022). A Dynamic-Model-Based Fault Diagnosis Method for a Wind Turbine Planetary Gearbox Using a Deep Learning Network. Prot. Control Mod. Power Syst..

[5] Han S., Feng Z., Zhang Y., Du M., Yang Y. (2024). Intelligent Fault Diagnosis of Planetary Gearbox Across Conditions Based on Subdomain Distribution Adversarial Adaptation. Sensors.

[6] Zhang C., Wei J., Niu R., Hou S., Zhang S. (2023). Similarity and Experimental Prediction on Load Sharing Performance of Planetary Gear Transmission System. Mech. Mach. Theory.

[7] Liu J., Li X.B., Xia M. (2023). A Dynamic Model for the Planetary Bearings in a Double Planetary Gear Set. Mech. Syst. Signal Process..

[8] Zhou X., He S., Dong L., Atluri S.N. (2022). Real-Time Prediction of Probabilistic Crack Growth with a Helicopter Component Digital Twin. AIAA J..

[9] Lee S.-H., Lee D.-W., Song H.-S., Jeong S., Ji Y., Song J.-S., Kim J., Yi B.-J. (2023). Robotic Manipulation System Design and Control for Non-Contact Remote Diagnosis in Otolaryngology: Digital Twin Approach. IEEE Access.

[10] Tuegel E.J., Ingraffea A.R., Eason T.G., Spottswood S.M. (2011). Reengineering Aircraft Structural Life Prediction Using a Digital Twin. Int. J. Aerosp. Eng..

[11] Florescu A. (2024). Digital Twin for Flexible Manufacturing Systems and Optimization through Simulation: A Case Study. Machines.

[12] Cunha C.D., Cardin O., Gallot G., Viaud J. (2021). Designing the Digital Twins of Reconfigurable Manufacturing Systems: Application on a Smart Factory. IFAC-PapersOnLine.

[13] Chang W., Sun W., Chen P., Xu H. (2025). Construction and Validation of a Digital Twin-Driven Virtual-Reality Fusion Control Platform for Industrial Robots. Sensors.

[14] Zhang J., Li C., Deng C., Luo T., Deng R., Luo D., Tao G., Cao H. (2025). Toward Digital Twins for Intelligent Manufacturing: Self-Adaptive Control in Assisted Equipment through Multi-Sensor Fusion Smart Tool Real-Time Machine Condition Monitoring. J. Manuf. Syst..

[15] Costa D., Pereira D., Brochado Â.F., Rocha E.M. (2025). Low-Cost and Non-Intrusive Human Digital Twin Component for Task and Navigation Tracking through Pose Estimation. CIRP J. Manuf. Sci. Technol..

[16] Rosen R., von Wichert G., Lo G., Bettenhausen K.D. (2015). About the Importance of Autonomy and Digital Twins for the Future of Manufacturing. IFAC-PapersOnLine.

[17] Haag S., Anderl R. (2018). Digital Twin—Proof of Concept. Manuf. Lett..

[18] Chakraborty S., Adhikari S., Ganguli R. (2021). The Role of Surrogate Models in the Development of Digital Twins of Dynamic Systems. Appl. Math. Model..

[19] Battula S., Alla N.S., Ramana V.E., Kumar N.K., Murthy S.B. (2024). Uncertainty Quantification for Digital Twins in Smart Manufacturing and Robotics: A Review. J. Phys. Conf. Ser..

[20] Wang S., Lai X., He X., Qiu Y., Song X. (2022). Building a Trustworthy Product-Level Shape-Performance Integrated Digital Twin with Multifidelity Surrogate Model. J. Mech. Des..

[21] Li X., Wu S., Gu Z., Zhang C., Sun W., Song X. (2021). Designing a Shape–Performance Integrated Digital Twin Based on Multiple Models and Dynamic Data: A Boom Crane Example. J. Mech. Des..

[22] Civera M., Surace C. (2022). Non-Destructive Techniques for the Condition and Structural Health Monitoring of Wind Turbines: A Literature Review of the Last 20 Years. Sensors.

[23] Guan G., Ye T., Liang G. (2025). Multi-Objective Optimization of a Fishery Administration Vessel’s Stern Flap Design Based on Surrogate Model. Ocean Eng..

